# Risk prediction and risk factor analysis of urban logistics to public security based on PSO-GRNN algorithm

**DOI:** 10.1371/journal.pone.0238443

**Published:** 2020-10-05

**Authors:** Mingjing Zhao, Shouwen Ji, Zhenlin Wei

**Affiliations:** School of Traffic and Transportation, Beijing Jiaotong University, Beijing, China; Tongii University, CHINA

## Abstract

For the complicated operation process, many risk factors, and long cycle of urban logistics, it is difficult to manage the security of urban logistics and it enhances the risk. Therefore, to study a set of effective management mode for the safe operation of urban logistics and improve the risk prediction mechanism, is the primary research item of urban logistics security management. This paper summarizes the risk factors to public security in the process of urban logistics, including pick up, warehouse storage, transport, and the end distribution. Generalized regression neural network (GRNN) is combined with particle swarm optimization (PSO) to predict accidents, and the Apriori algorithm is used to analyze the combination of high-frequency risk factors. The results show that the method of combining GRNN with PSO is effective in accident prediction and has a powerful generalization ability. It can prevent the occurrence of unnecessary urban logistics public accidents, improve the ability of relevant departments to deal with emergency incidents, and minimize the impact of urban logistics accidents on social and public security.

## Introduction

Taniguchi et al. [1] defines urban logistics as "the process by which private enterprises achieve the overall optimization of logistics and transportation activities in the market economy, taking into account the urban traffic environment, traffic congestion, and energy consumption".

The city is the concentration of logistics activities, the logistics activities in the city are the most important part of the whole logistics link. The risk of urban public security refers to the force majeure and the possibility of aim existence that pose a threat to the basic values, norms, and interests of the urban public sphere [2].

To ensure logistics activities operation smoothly, meet people's production and living needs, and minimize the impact to public security on the premise of the overall coordinated and sustainable development of the city, this paper proposes a set of risk analysis and prediction model of urban logistics to public security.

As the major artery in the national economic system, the logistics system provides security for the national economy and social life. In recent years, with the frequent occurrence of public accidents in the urban logistics industry, China's logistics industry loses about 15 billion yuan because of packaging accidents every year. The loss caused by loading, unloading, and transportation accidents is about 50 billion yuan. The loss caused by the storage accident is about 3 billion yuan [3]. This has caused the academic circle to the city logistics public security question discussion. How to construct a safe and efficient urban logistics system and predict the public security risk of urban logistics is a problem worth discussing.

This paper studies the impact of urban logistics to public security. The operation process of urban logistics is complicated, including transportation, storage, terminal distribution, and other links of goods. There are many risk factors to urban public security, posing a threat to public security. The public safety accidents caused by urban logistics studied in this paper mainly include vehicle collision, vehicle explosion and fire, warehouse explosion and fire, the contraband enters the delivery channel, and home invasion robbery by couriers. In recent years, with the rapid development of e-commerce, the development process of urban logistics has been accelerated, and related public accidents have occurred frequently, causing substantial loss of personnel and property. At present, there is still much room for improvement in the study of urban logistics on the analysis of public security risk factors and risk prediction. Analysis of risk factors is the primary link of risk management, which can understand the principal causes of accidents and high-frequency combination factors, and provide important information for risk prevention. Risk level prediction can prevent and control risks in urban logistics operations and timely take the corresponding management and measures. It has important research significance.

Scholars' research on accident risk is generally concentrated in the fields of coal mine, transportation, aviation and so on. The commonly used risk research methods include Logit model [4], negative binomial models [5], BP neural network [6], grey system theory [7], fuzzy comprehensive evaluation method [8], analytic hierarchy process [9, 10], Bayesian network [11, 12], spatial models [13, 14], etc., focusing on the evaluation of research risk. The research on risk prediction mainly focuses on the fields of transportation [15], coal mine [16], earthquake [17], medicine [18], economy [19], computer [20], civil engineering [21] and so on. The main prediction methods are regression, Bayesian network, Artificial neural networks, Support Vector Machine, random forest, etc. In recent years, with the increase of logistics accidents, scholars pay more and more attention to the research of logistics accident prediction. Ke Li [22] et al. used a factor analysis method to determine the factors affecting the risk of emergency logistics, and established Bayesian network to predict and control the risk of emergency logistics, providing a new way to avoid the risk of emergency logistics. Aiming at the uncertainty and complexity of urban logistics system, Mingjing Zhao [23] et al. adopted the method of combining interpretive structure model and Bayesian network model to analyze logistics risk factors and find out the sensitive nodes of logistics system. Weiyang Xu [24] et al. proposed an improved neural network model to realize the analysis of various cold chain logistics indicators and the comprehensive risk prediction of cold chain logistics given the shortcomings of cold chain logistics risk management. Dafeng Xu [25] et al. proposed an alternative model based on particle swarm optimization (PSO), which proved that PSO could predict logistics risks more accurately by comparing its performance with that of the fuzzy comprehensive evaluation method. However, most of the risk factors in these methods are independent of each other. In reality, these factors are often interrelated.

Based on the above problems, this paper adopts the method of literature review and the investigation of transportation companies to divide the factors affecting the risk of urban logistics into five dimensions: people, vehicles and facilities, goods, management, and weather, including 11 factors. BP neural network, GRNN, and PSO-GRNN are used to predict the accident level, and the results show that PSO-GRNN can effectively improve the accuracy of accident level prediction reaching 80%, which provides a new idea for the study of urban logistics safety management and has a good reference significance. Based on the analysis of high-frequency influencing factors, the results show that there are 7 single factors, 10 two-factor combinations, 6 three-factor combinations and 1 four-factor combination that are closely related to accidents, and safety managers need to pay special attention to them. Take precautions in advance to reduce the incidence of urban logistics accidents.

The rest of this article is structured as follows. In the second section, we introduce several algorithms used in this paper. The third section is the comparison of the prediction results of several models and the analysis of high-frequency risk factors. The fourth section is the discussion of the results.

## Methodology

### Generalized regression neural network (GRNN)

Generalized regression neural network is an artificial neural network technology. Dr. D. f. Specht [26] proposed it in 1991 and is another variant form of radial basis neural network. Based on non-parametric regression, GRNN performs Parzen non-parametric estimation with the sample data as the posterior condition and calculates the network output according to the principle of maximum probability. GRNN has good nonlinear mapping ability and learning speed, and can solve classification problems. When the sample data is small, it has a beneficial prediction effect. So far, GRNN has been widely used in mechanical engineering [27], clinical medicine [28], electrical science [29], and other disciplines and engineering fields.

**Fig 1:**
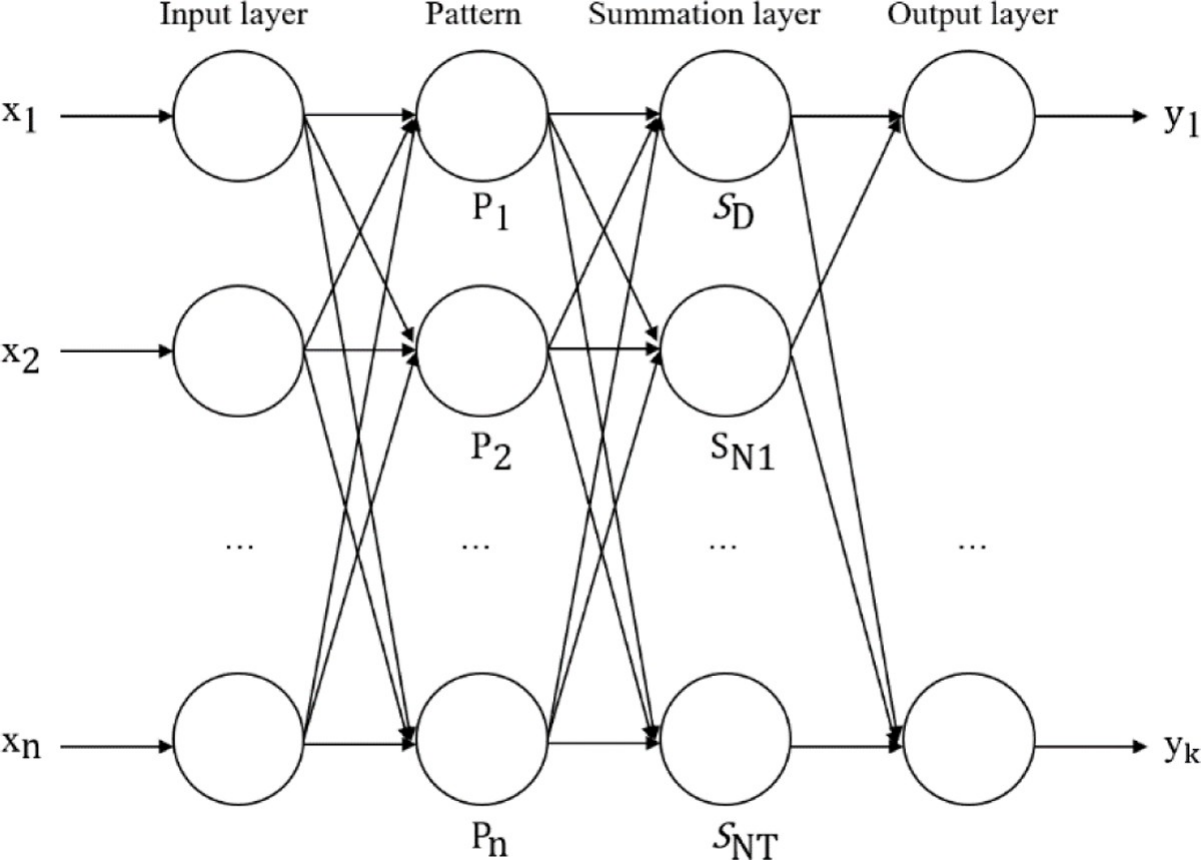
Generalized regression neural network structure diagram.

The generalized regression neural network comprises four layers, as shown in Fig 1, namely the input layer, pattern layer, summation layer, and output layer.

The prediction process of GRNN model is:

Suppose that the joint probability density of the two random variables *x* and *y* is *f*(*x*, *y*). If the observed value of *x* is known be *x*_0_, the regression of *y* to *x* is shown in formula (1):

$$E(y|x_{0})=(x_{0})=\frac{\int_{-\infty }^{0}yf(x_{0},y)dy}{\int_{-\infty }^{0}f(x_{0},y)dy}$$ (1)

*y*(*x*_0_) is the predicted output of *y* with input *x*_0_. By applying Parzen non-parametric estimation, the sample data set ${\left\{x_{i},y_{i}\right\}}_{i=1}^{n}$ can estimate the density function *f*(*x*_0_, *y*) according to Eqs (2) and (3):

$$f(x_{0},y)=\frac{1}{n{\left(2\pi \right)}^{\frac{p+1}{2}}\sigma_{}^{p+1}}\sum_{i=1}^{n}e^{-d(x_{0},x_{i})}e^{-d(x_{0},x_{i})}$$ (2)

$$d(x_{0},x_{i})=\sum_{j=1}^{p}{\left[(x_{0j}-x_{ij})/\sigma \right]}^{2},d(y,y_{i})={\left[y-y_{i}\right]}^{2}$$ (3)

Where *n* is the sample size, *p* is the dimension of the random variable *x*, and σ is called the smooth factor. According to Eqs (2), (3) and (4) can be obtained as follows:

$$y(x_{0})\frac{\sum_{i=1}^{n}\left(e^{-d(x_{0},x_{i})}\int_{-\infty }^{+\infty }ye^{-d(y_{0},y_{i})}dy\right)}{\sum_{i=1}^{n}\left(e^{-d(x_{0},x_{i})}\int_{-\infty }^{+\infty }e^{-d(y_{0},y_{i})}dy\right)}$$ (4)

Since $\int_{-\infty }^{+\infty }xe^{-x^{2}}dx=0$. Simplified Eqs (4) and (5) can be obtained as follows:

$$y(x_{0})\frac{\sum_{i=1}^{n}ye^{-d(y_{0},y_{i})}}{\sum_{i=1}^{n}e^{-d(x_{0},x_{i})}}$$ (5)

In the GRNN model, the smooth factor σ has a significant influence on the prediction performance of the network. The larger the value of σ, the greater the error of the prediction result, and the smaller the value of σ, the more accurate the prediction result. When the value of σ approaches 0, over-fitting is easy to occur. Therefore, the prediction accuracy of the model is largely influenced by σ. In this paper, an improved PSO algorithm is used to optimize σ value.

### Particle swarm optimization (PSO)

Particle swarm optimization (PSO) was proposed in 1995 by American electrical engineer Eberhart and social psychologist Kenndy. The algorithm is based on the foraging behavior of birds. Compared with other intelligent algorithms, the PSO algorithm has the advantages of convenient implementation, fast convergence speed, and less parameter setting. It has been used in computer [30], civil engineering [31], clinical medicine [32] and other fields.

**Fig 2:**
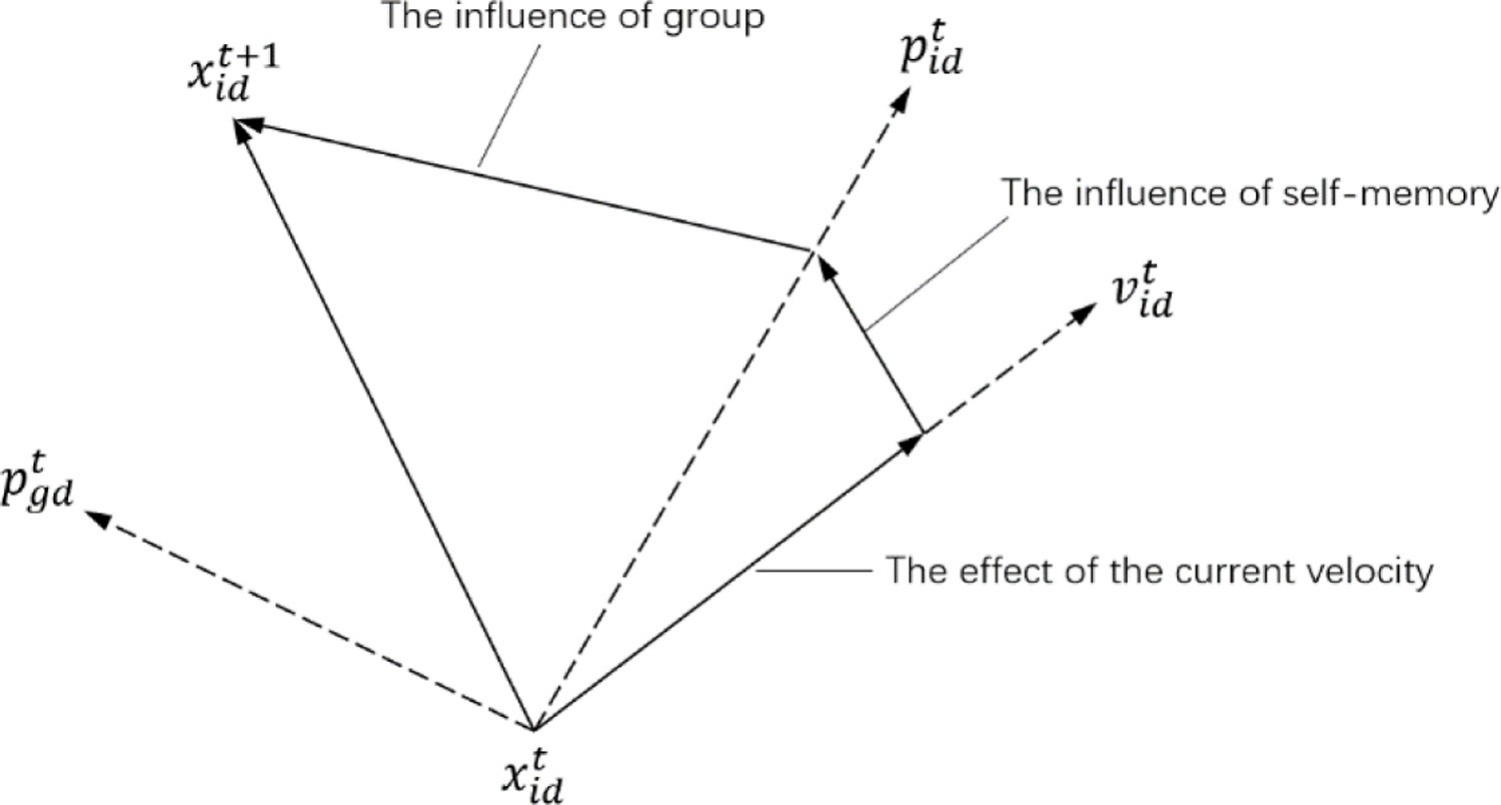
The updated pattern of particle positions for each generation.

In the PSO algorithm, the potential solution of each optimization problem is a particle in the search space. All particles have a fitness value determined by an optimized function, and each particle also has a speed that determines their flight direction and distance. Fig 2 shows the update mode of particle position. *x* is the starting position of the particle, *v* is the velocity of the particle, and *p* is the optimal position of the searched particle.

The calculation steps of PSO are as follows.

Step 1: initialize the particle swarm. Set the population size *N*, the position of each particle *x*_*i*_, and the velocity *v*_*i*_.

Step 2: calculate individual extremum and global optimal solution. Calculate the fitness value *Fit*_*i*_ of each particle. For each particle, compare its fitness value *Fit*_*i*_ with the individual extreme value pbest(*i*). If *Fit*_*i*_>pbest(*i*), replace pbest(*i*) with *Fit*_*i*_ Compare *Fit*_*i*_ with the global extreme value gbest and replace gbest with *Fit*_*i*_ if *Fit*_*i*_>gbest.

Step 3: update speed and location. The position *x* and velocity *v* of the particle are updated according to Eqs (6) and (7).

$$v_{id}=\omega *v_{id}+c_{1}r_{1}(p_{id}-x_{id})+c_{2}r_{2}(p_{gd}-x_{id})$$ (6)

$$x_{id}=x_{id}+v_{id}$$ (7)

Where, *c*_1_, *c*_2_ is the learning factor, also known as acceleration constant. *r*_1_, *r*_2_ is the uniform random number within the range of [0,1]. *ω* is the inertia weight.

Step 4: termination conditions. Exit the loop if the termination condition is met, otherwise return to step 2. There are two kinds of termination conditions, one is the maximum algebra, the other is the error within the specified range.

### Improved PSO algorithm

PSO algorithm has the advantages of easy to use and fast convergence. However, when the search environment is complex, PSO tends to fall into local optimality, resulting in low convergence accuracy and difficulty in convergence. Inertia weight *ω* has the balance function of maintaining global and global search capability. When *ω* is larger, it is beneficial to improve the convergence speed of the algorithm. When *ω* is relatively small, it is beneficial to improve the convergence accuracy of the algorithm, but it is easy to fall into the local optimal. Therefore, this paper adopts a strategy of adaptive adjustment, as shown in Eq (8), the value of *ω* decreases gradually as the iteration progresses.

$$\omega =\{\begin{array}{cc} \omega_{\min}-\frac{\left(\omega_{\max}-\omega_{\min})*(f-f_{\min}\right)}{f_{avg}-f_{\min}} & ,f\leq f_{avg}\\ \omega_{\max} & ,f\leq f_{avg} \end{array}$$ (8)

### Apriori algorithm

Apriori is an algorithm for mining frequent item sets of Boolean association rules proposed by R. Agrawal and R. Srikant [33] in 1994. The basic idea of this algorithm is that all the item sets whose support degree is greater than the minimum support degree are called frequent item sets or frequency sets for short. All frequency sets are identified first, from which strong association rules are generated that must satisfy minimum support and minimum confidence. The found frequency set is then used to generate the desired rule, producing all the rules that contain only the items of the set. Once these rules are generated, it retains only those rules greater than the given minimum confidence. To generate all frequency sets, it uses the recursive method. Support degree *s* and confidence degree *c* are calculated as shown in Eqs (9) and (10).

$$s(X\to Y)=\frac{\sigma (X\cup Y)}{N}$$ (9)

$$c(X\to Y)=\frac{\sigma (X\cup Y)}{\sigma (X)}$$ (10)

### Model performance criteria

Based on the criteria of accuracy rate (ACC), the sum of squares due to error (SSE), mean squared error (MSE), coefficient of determination (R^2^)and other parameters, the performance analysis of BP, GRNN and PSO-GRNN models are evaluated:

The means square error (MSE) is shown in Eq (11):

$$\text{MSE}=\frac{1}{n}\sum_{i=1}^{n}{\left(\hat{y_{i}}-y_{i}\right)}^{2}$$ (11)

The coefficient of determination (R^2^) is shown in formula (12):

$$R^{2}=1-\frac{\sum_{i=1}^{n}{\left(y_{0}{}_{i}-y_{i}\right)}^{2}}{\sum_{i=1}^{n}{\left(y_{i}-\bar{y}\right)}^{2}}$$ (12)

The sum of squares due to error (SSE) is shown in Eq (13):

$$\text{SSE}=\sum_{i=1}^{n}{\left(\hat{y_{i}}-y_{i}\right)}^{2}$$ (13)

The prediction accuracy (ACC) is shown in Eq (14):

$$ACC=\frac{n_{correct}}{n}$$ (14)

## Risk prediction of urban logistics to public security

### Data preparation

The urban logistics system is a complex system with many links and factors. This paper analyzes the risk factors of urban logistics from the perspective of life cycle theory. The life cycle of urban logistics is a continuous process, so it is difficult to distinguish each stage accurately, and there are many overlaps between adjacent links. This paper identifies and analyzes risk factors in the process of pick up, warehouse storage, transport, and the end distribution, which may easily affect public security, as its life cycle. Based on literature review and field research, we construct the risk index system of urban logistics on public security, including 5 first-level indicators and **9** second-level indicators. The primary indicators include management (M), environment (E), human factors (H), transportation tools and facilities (F), and goods factors (G). Human factors include skill level (H1), physical condition (H2), safety awareness (H3), personnel quality (H4), transport vehicle (F1), maintenance and inspection (F2), facility instrument (F3), and goods category (G1) and storage issues (G2). System failure is evaluated with 5 grades: [0.0.2] interval shows serious risk; (0.2,0.4] shows a greater risk; (0.4,0.6] shows slight risk; (0.6,0.8] shows risk signs; (0.8,1.0] shows no risk. Table 1 shows the details.

**Table 1:** Direct factors involved in urban logistics to public safety accidents.

Direct factors	Description	Value set
Management(M)	Manage personnel, facilities and storage	Normal(0.8,1.0], Abnormal[0,0.8]
Environment(*E*)	Weather, roads, etc	Normal(0.8,1.0], Abnormal[0,0.8]
*Human(H)*		
Skill level(*H*_1_)	Skill, experience	Normal(0.8,1.0], Abnormal[0,0.8]
Physical condition(*H*_2_)	Sickness, fatigue	Normal(0.8,1.0], Abnormal[0,0.8]
Safety awareness(*H*_3_)	Safety awareness	Normal(0.8,1.0], Abnormal[0,0.8]
Personnel quality(*H*_4_)	Quality, follow rules	Normal(0.8,1.0], Abnormal[0,0.8]
*Transportation tools and facilities(F)*		
Transport vehicle(*F*_1_)	Vehicle performance, type	Normal(0.8,1.0], Abnormal[0,0.8]
Maintenance and inspection(*F*_2_)	Maintenance and inspection regularly	Normal(0.8,1.0], Abnormal[0,0.8]
Facility instrument(*F*_3_)	Fully equipped	Normal(0.8,1.0], Abnormal[0,0.8]
*Goods factor(G)*		
Goods category (*G*_1_)	Category risk	Normal(0.8,1.0], Abnormal[0,0.8]
Storage issues(*G*_2_)	Store as required	Normal(0.8,1.0], Abnormal[0,0.8]

We collected 235 from public accidents caused by urban logistics in China in the past ten years [34–36]. Referring to the classification standard of accidents in the regulations on the reporting, investigation, and handling of production safety accidents in China, and combining with the actual situation of urban logistics. We can divide accidents into five levels: Ⅴ major accident (over 10 dead or over 50 seriously injured or over 50 million RMB loss), Ⅳ larger accident (3–9 dead or 10–49 seriously injured or 10–50 million RMB lost), Ⅲ fatalities (below 3 people dead or serious injury or loss of less than 10 million RMB), Ⅱ serious injury accident (only seriously no death or loss of RMB 30–10 million), Ⅰ minor accidents (only minor injuries or less than 300000 RMB lost). S1 Table shows the accident data set.

### The risk prediction model of urban logistics to public security

In this paper, we use BP neural network, GRNN, and GRNN-PSO to predict the risk level of urban logistics to public security.175 samples are randomly selected for model training, and the other 60 samples are tested and verified and compare the predicted results.

#### BP neural network prediction model

BP neural network is a multilayer feedforward network which is trained by error back propagation algorithm. It consists of the input layer, the hidden layer and the output layer. The hidden layer can be one or more layers, and the different layers respectively include multiple parallel neuron signals. In this paper, the risk values of 11 risk factors are taken as input, so the input layer is 11 neurons. The output is the level of risk, so the output layer is 1 neuron. This model adopts a single hidden layer structure and the steepest descent method for training. In the process of model training, we select the number of hidden neurons as 1, 4, 7, 10, 13, and 16 [37] for comparison to find the optimal model. In the process of training, the training algorithm adjusts the weight and deviation iteratively to make the predicted value closer to the actual value.

**Fig 3:**
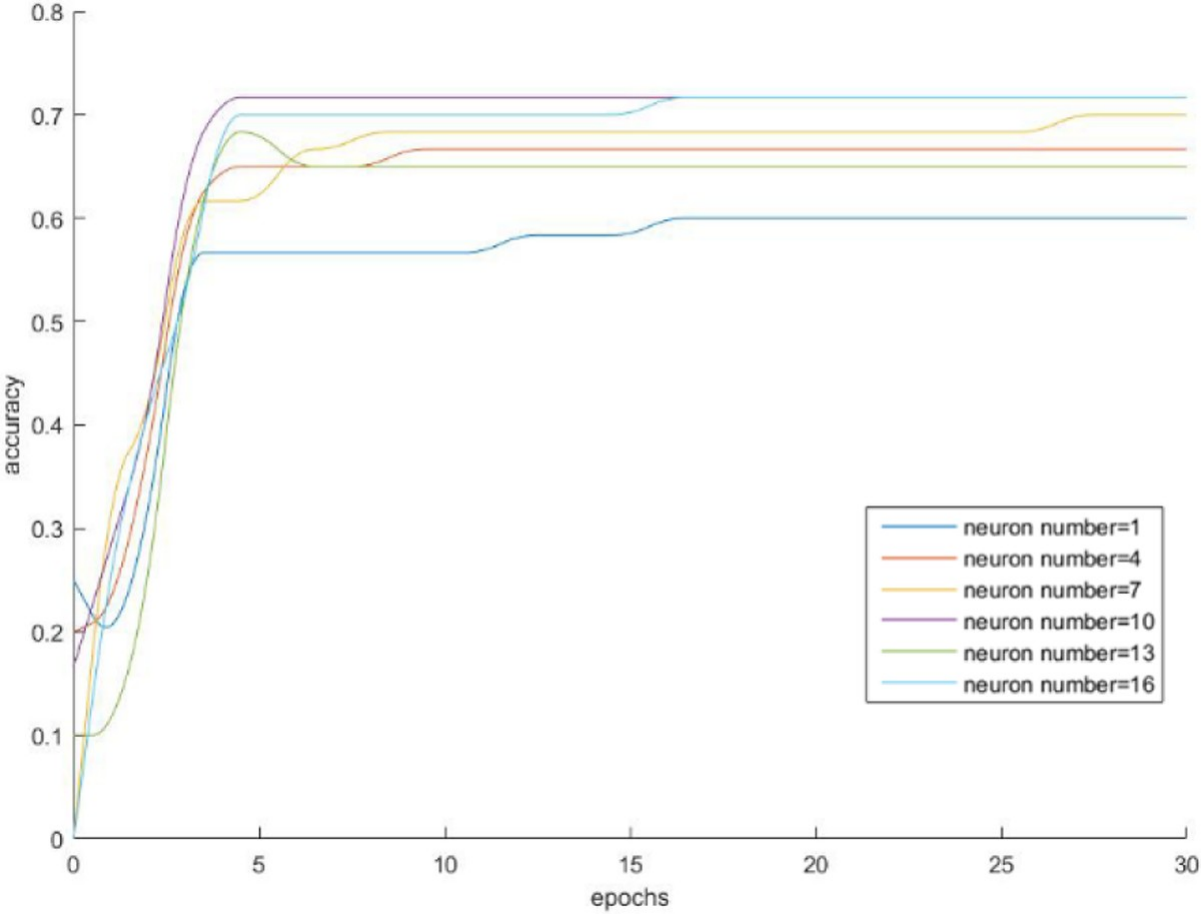
Training process with the different number of hidden layer neurons.

**Fig 4:**
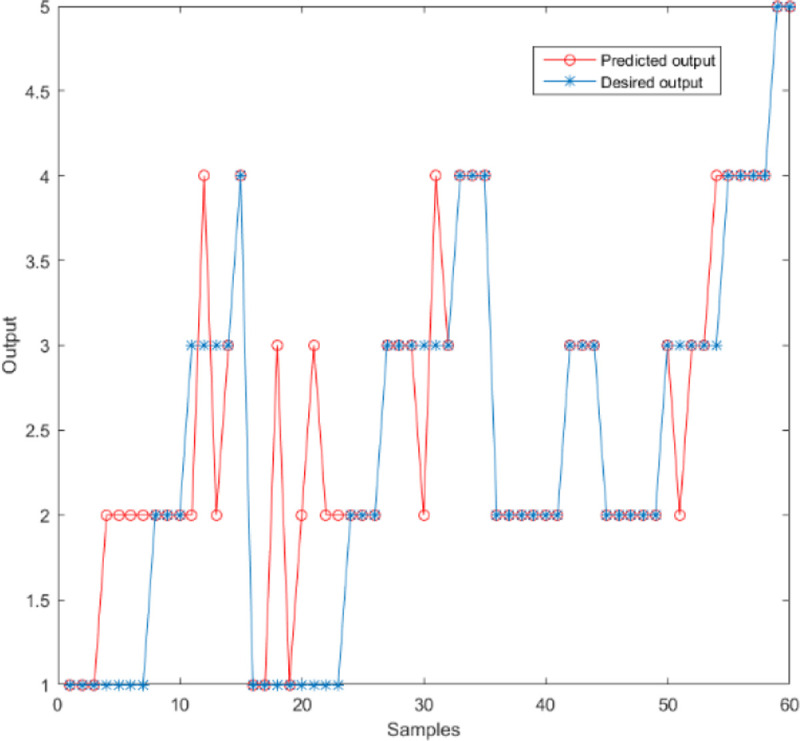
Output results when the number of neurons in the hidden layer is 7.

Fig 3 shows the BP neural network training process with the different number of hidden layers of neurons, and it achieves the convergence effect in the fifth training. The prediction accuracy, SSE, MSE, and R^2^ of a different number of neurons are shown in Table 2. The prediction accuracy is highest when the number of hidden layer neurons is 10. The hidden layer neuron is 7, the error sum squared is minimum. Fig 4 shows the output at this point.

**Table 2:** The prediction performance index of BP neural network with a different number of hidden layer neurons.

Number of neurons	ACC	SSE	MSE	R^2^
1	58.33%	28.002	0.4667	0.82603
4	66.67%	19.998	0.3333	0.85885
7	68.33%	16.998	0.2833	0.90965
10	73.33%	19.002	0.3167	0.8841
13	65.00%	33	0.55	0.8843
16	71.67%	31.002	0.5167	0.89347

#### GRNN prediction model

**Fig 5:**
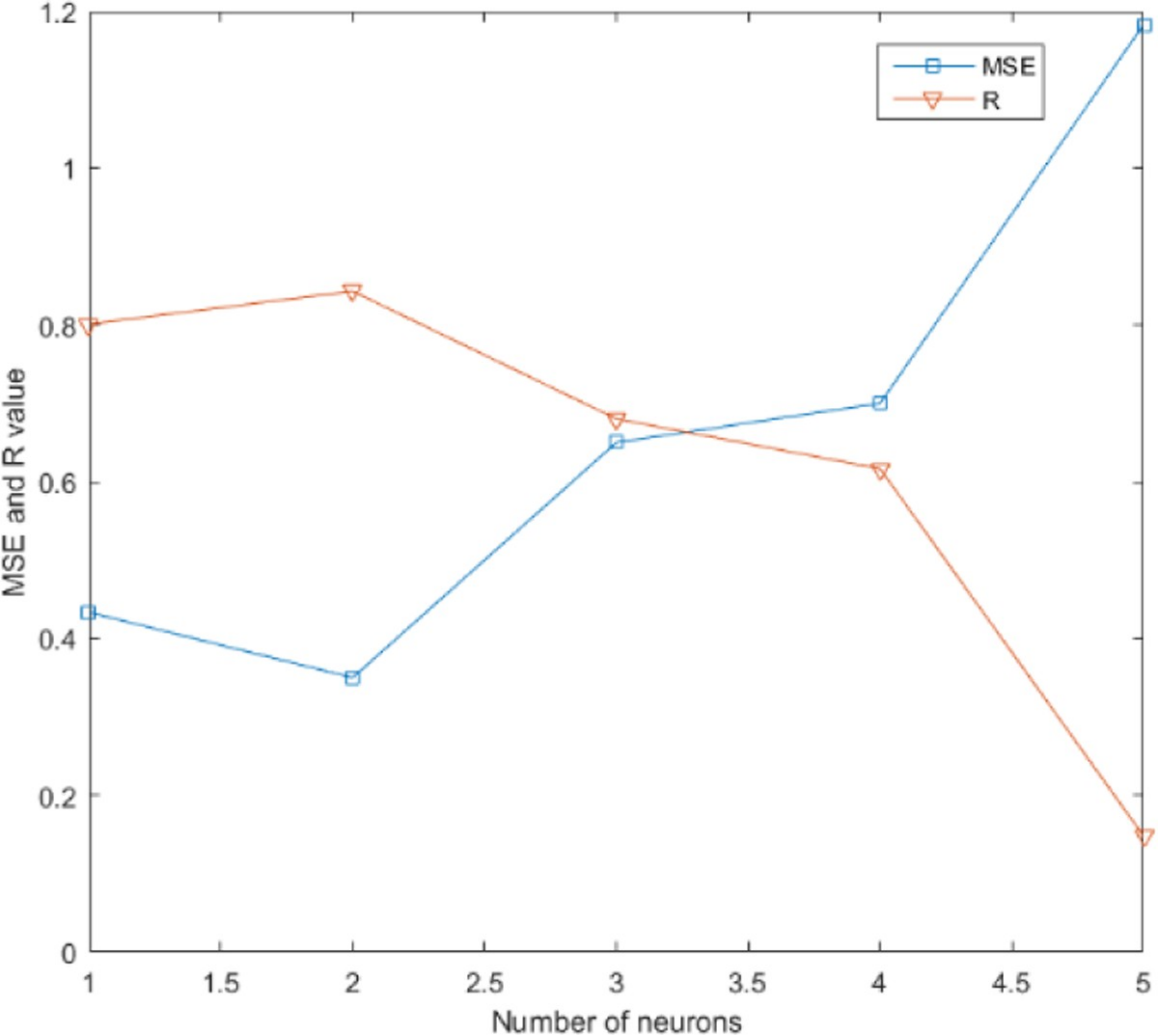
The values of MSE and R^2^ vary with different smooth factors.

**Fig 6:**
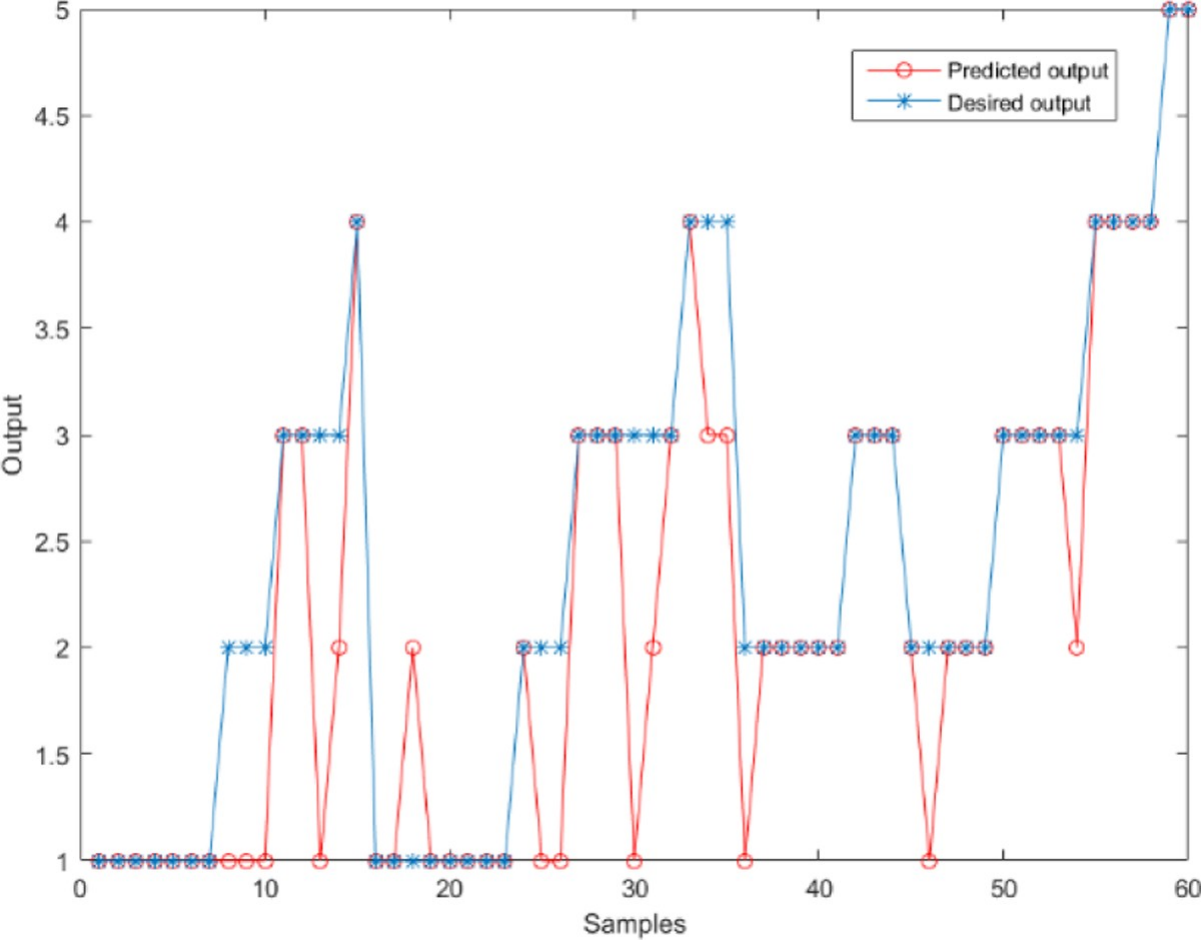
The comparison between the predicted results, and the expected results when the smoothing factor is 0.5.

In the GRNN structure, the regression analysis of the input variable *x* is the calculation of the output *y* with the maximum probability value. The only parameter to be learned is the width coefficient of the Gaussian function, which is the smooth factor σ. The training aim is to optimize the parameters and minimize the mean square error of the predicted values. The GRNN model uses the same sample data as the BP neural network model for prediction, and the smooth factor is set between 0 and 2 for prediction. We compared the results of smooth factors 0.3, 0.5, 0.7, 1.0, and 1.2. The prediction accuracy, SSE, MSE, and R^2^ are shown in Table 3. It is found from Table 3 that the prediction results when the smooth factor is 0.5 are relatively satisfactory. Compared with other values, the error is the smallest and the R Square is the highest. The accuracy of prediction is 75%. Fig 5 shows the comparison of the sum of squares of errors and the R Square. Fig 6 shows the comparison between the predicted results and the expected results when the smooth factor is 0.5.

**Table 3:** The prediction performance index of GRNN with different smooth factor.

Smooth factor	ACC	SSE	MSE	R^2^
0.3	76.67%	25.998	0.4333	0.8011
0.5	75.00%	21	0.35	0.8430
0.7	65%	39	0.65	0.6802
1.0	60%	42	0.7	0.6165
1.2	55%	70.998	1.1833	0.1477

#### PSO-GRNN prediction model

**Fig 7:**
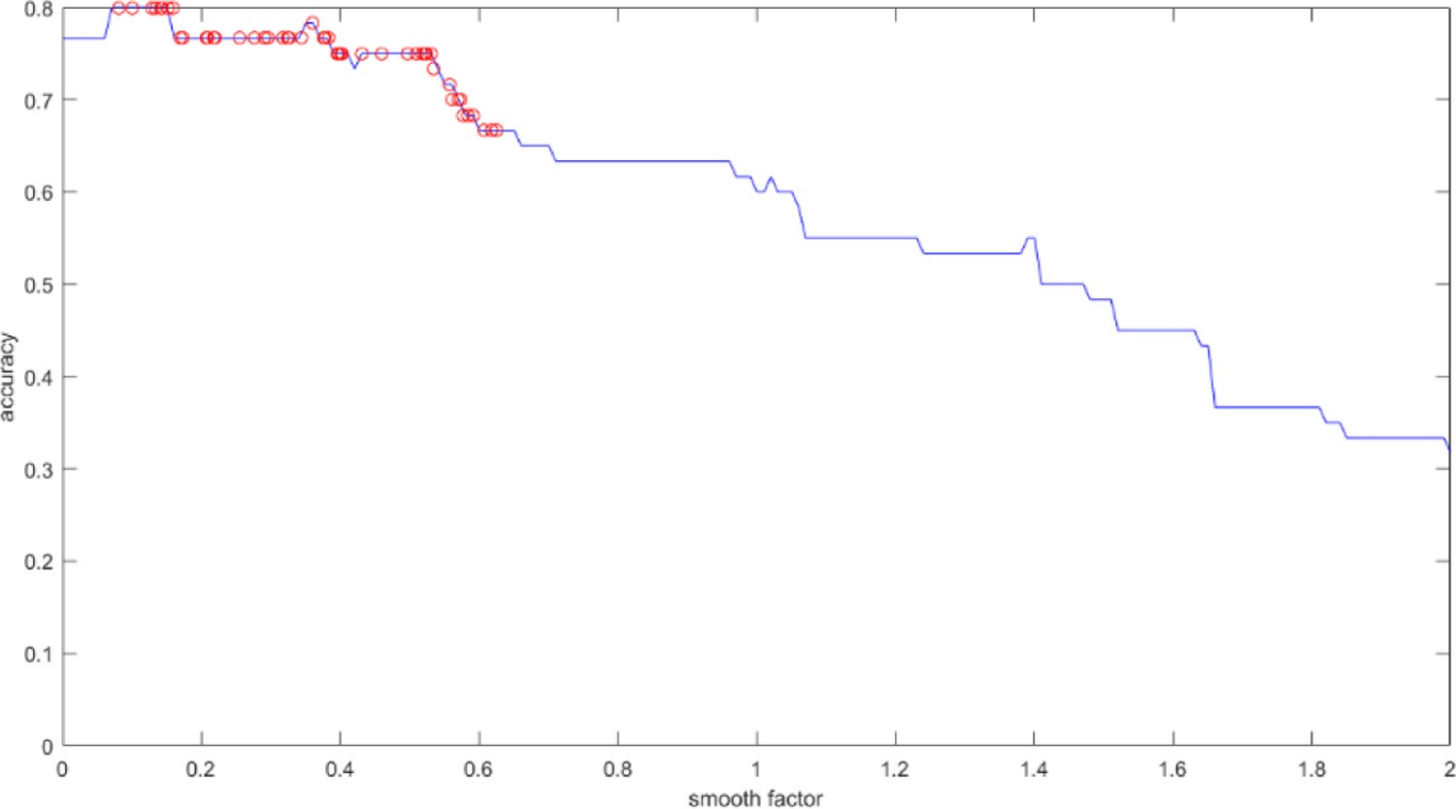
The optimal trajectory of the particle.

**Fig 8:**
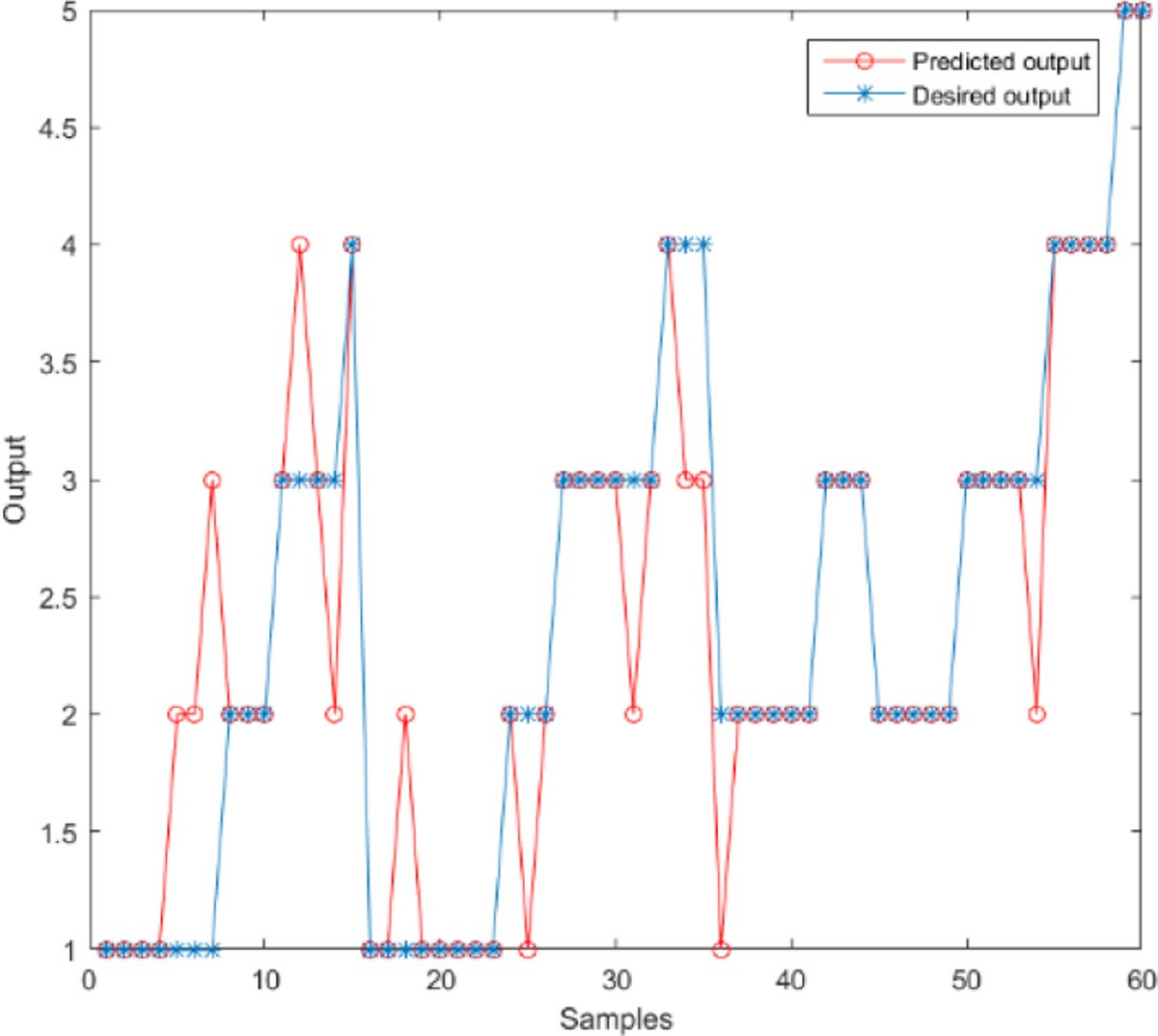
The comparison between the predicted results and the desired results.

GRNN can get different prediction results by using different smooth factors. This is very similar to the PSO algorithm in which the particle moves towards the target by modifying its speed and position. PSO algorithm is used to find the optimal smooth factor in the GRNN model, and we obtain the optimal prediction result. The particle position is limited to [0,2], and the position of each particle represents the current set of smooth factors in the GRNN model. Taking the output accuracy of the training sample set as an adaptation function, the higher the prediction accuracy is, the better the particle performance in the search is. The speed limit is [-1,1], the inertia weight is 0.5, and both the self-learning factor and the group learning factor are set to 0.5. Fig 7 shows the optimal trajectory of the particle. The optimal smooth factor is 0.14319, the accuracy is 80%, the mean square error is 0.25, and the determination coefficient is 0.8907. Fig 8 shows the comparison between the predicted results and the desired results.

#### Comparative analysis of the prediction results of the three models

In this paper, BP, GRNN, PSO-GRNN are selected to predict the level of urban logistics accidents. The hidden layer of BP neural network consists of 10 neurons, which are trained by the steepest descent method. The smooth factor of GRNN model is set to 0.5. In PSO-GRNN model, the particle velocity limit is [-1,1], the inertia weight is 0.5, and the self-learning factor and group learning factor are set to 0.5. To compare the accuracy of the three methods in predicting urban logistics accidents. Each model is run randomly 6 times. The results are recorded (Table 4) and the average values are calculated (Table 5) for performance comparison.

**Table 4:** Comparison of prediction results of different mathematical models.

Model	NO	ACC	SSE	MSE	R^2^
BP	1	63.33	22.002	0.3667	0.8348
	2	70	18	0.3	0.8672
	3	71.67	16.998	0.2833	0.8751
	4	66.67	22.998	0.3833	0.8265
	5	65	36	0.6	0.7099
	6	65	21	0.35	0.8430
GRNN	1–6	75.00%	21	0.35	0.8430
PSO-GRNN	1	76.67	22.998	0.3833	0.8265
	2	80%	15	0.25	0.8907
	3	78.33%	22.002	0.3667	0.8348
	4	80%	15	0.25	0.8907
	5	80%	15	0.25	0.8907
	6	76.67%	16.998	0.2833	0.8751

**Table 5:** The average of the results of different mathematical models.

Model	ACC	SSE	MSE	R^2^
BP	66.94%	22.833	0.381	0.826
GRNN	75.00%	21.000	0.350	0.843
PSO-GRNN	78.61%	17.833	0.297	0.868

**Fig 9:**
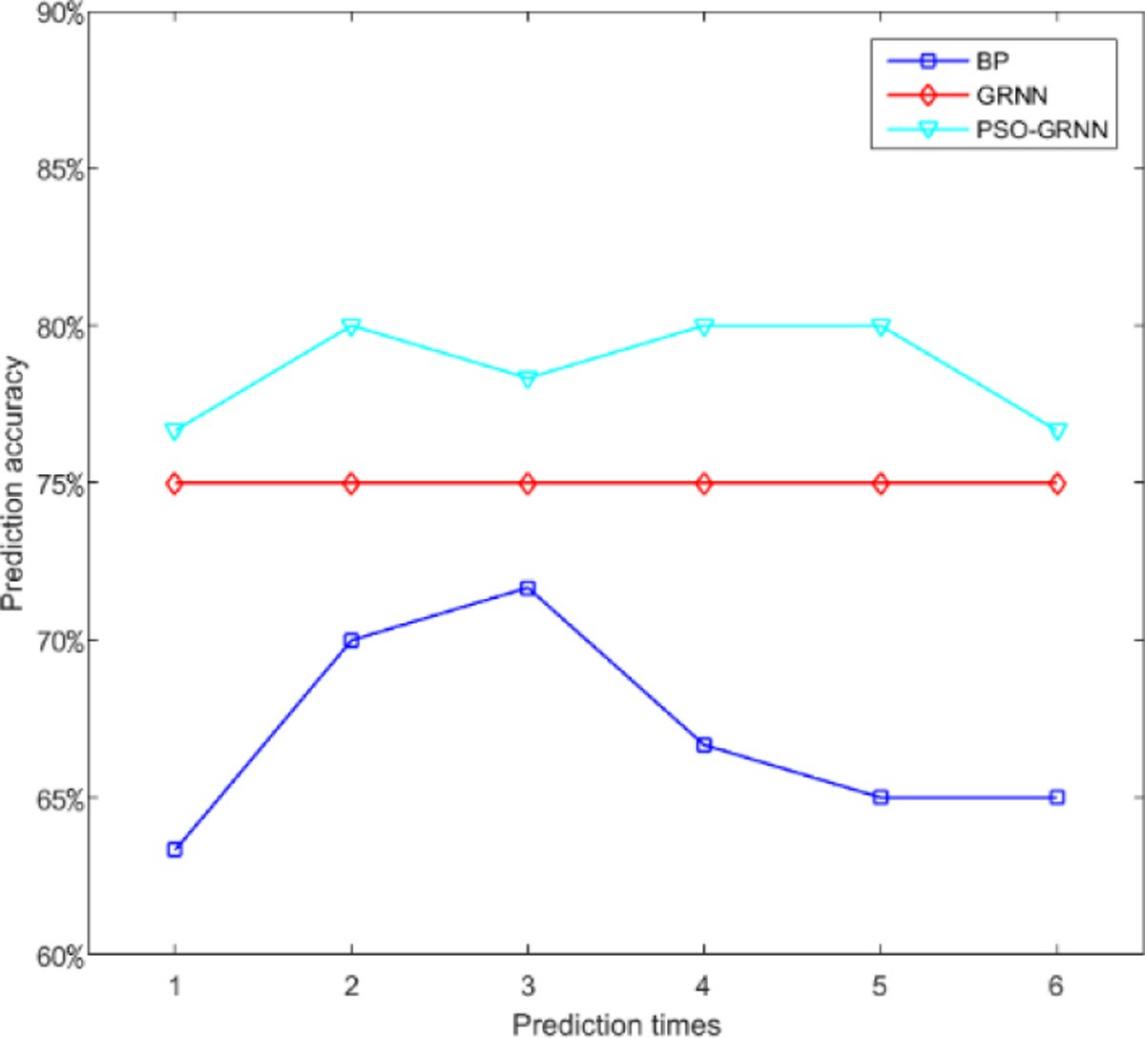
Comparison of prediction accuracy of the three models.

**Fig 10:**
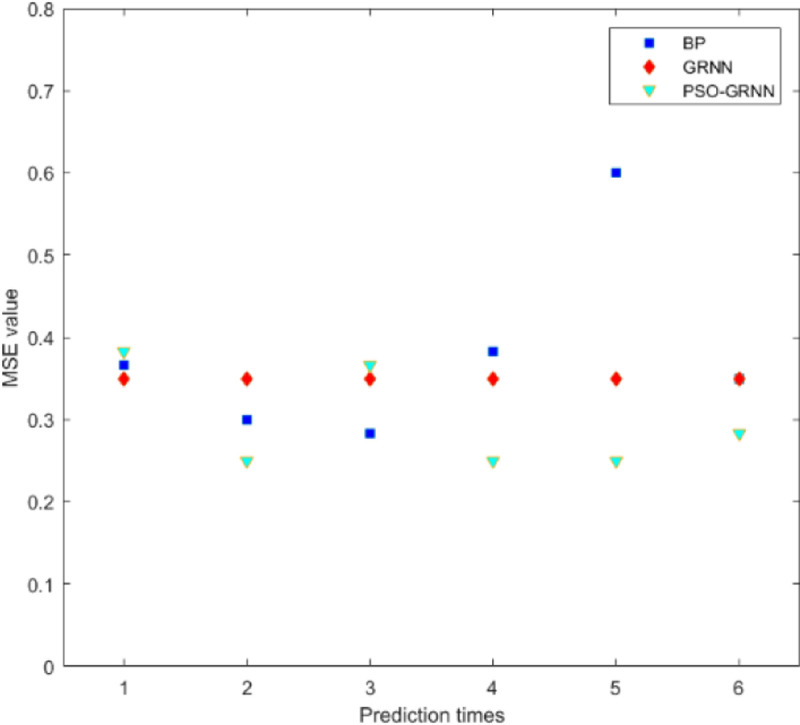
MSE comparison of the three models.

**Fig 11:**
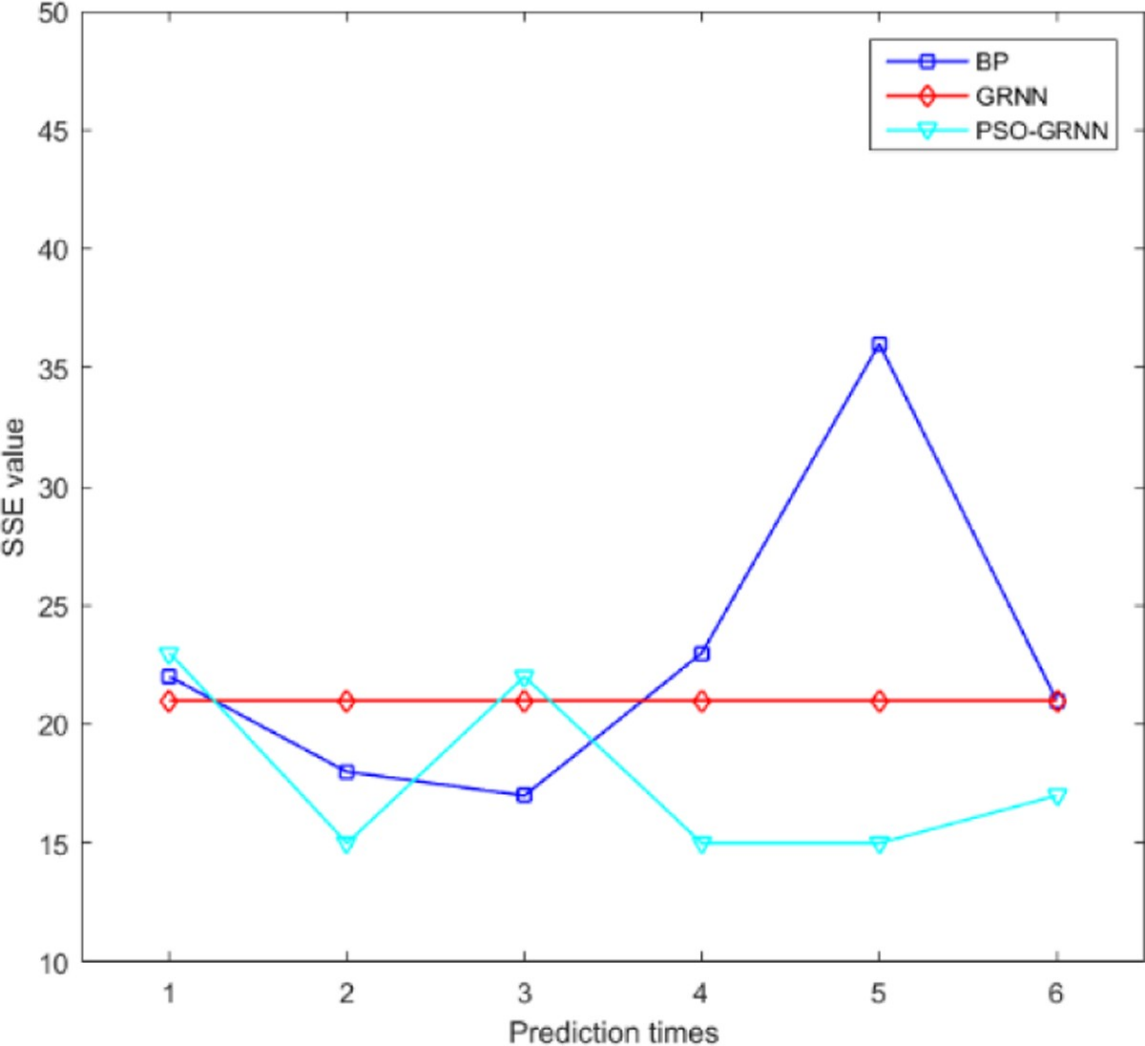
SSE comparison of the three models.

**Fig 12:**
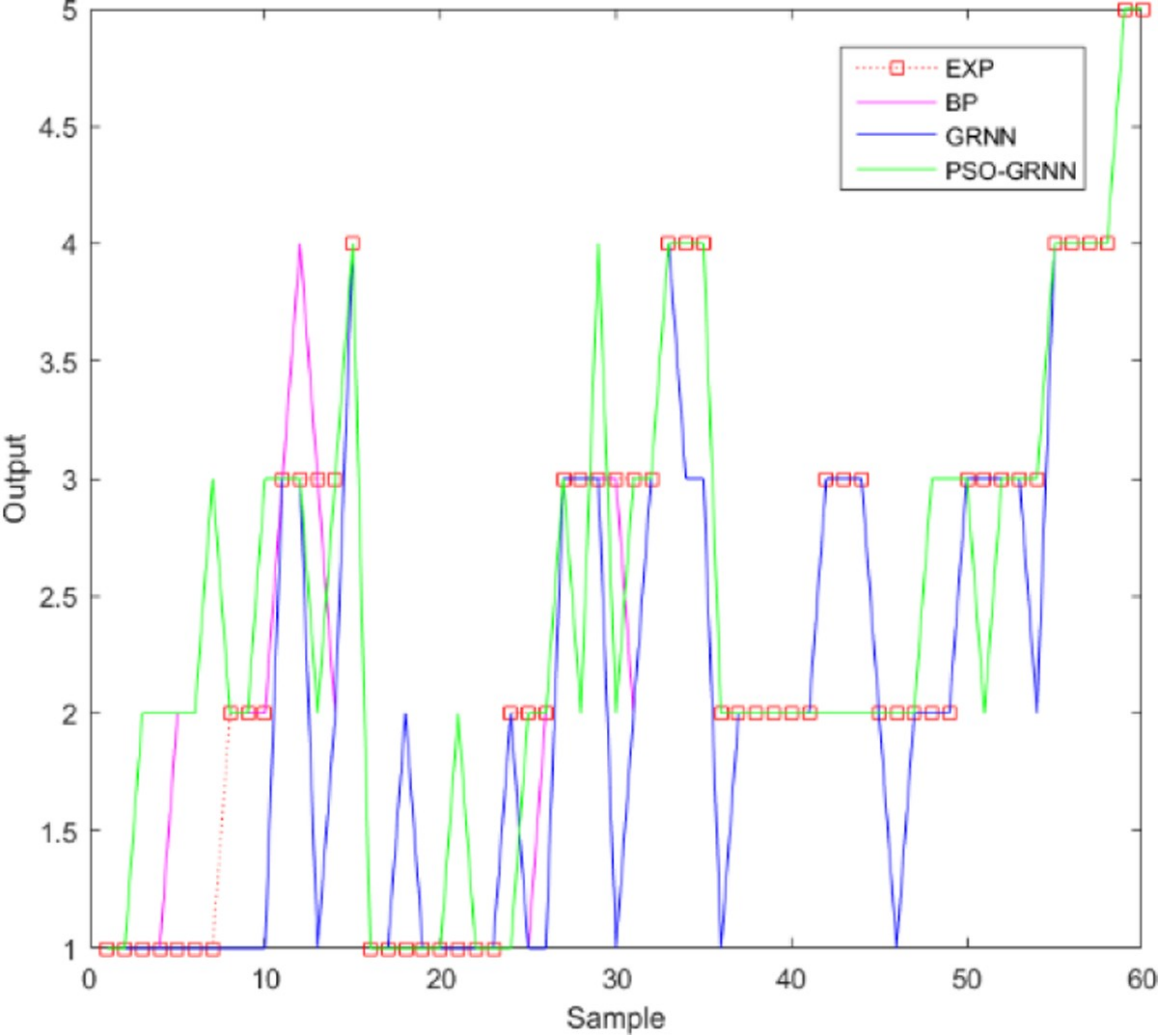
Comparison of the predicted results of the three models.

Fig 9 shows the comparison accuracy of the three models. Fig 10 shows the comparison of MSE. It shows SSE comparison in Fig 11. The lowest of SSE of BP, GRNN, and PSO-GRNN are 16.998, 21, and 15 respectively, showing that PSO-GRNN performs best in the risk level prediction. From the perspective of the prediction accuracy of risk level, the highest accuracy of BP, GRNN, and PSO-GRNN are 71.67%, 75.00%, and 80%, respectively. It can be seen that PSO-GRNN has the highest prediction accuracy among the three methods, and its prediction results are shown in Fig 12. To sum up, PSO-GRNN has the highest ACC and R, and the lowest SSE and MSE.

#### Sensitivity analysis of factors

**Fig 13:**
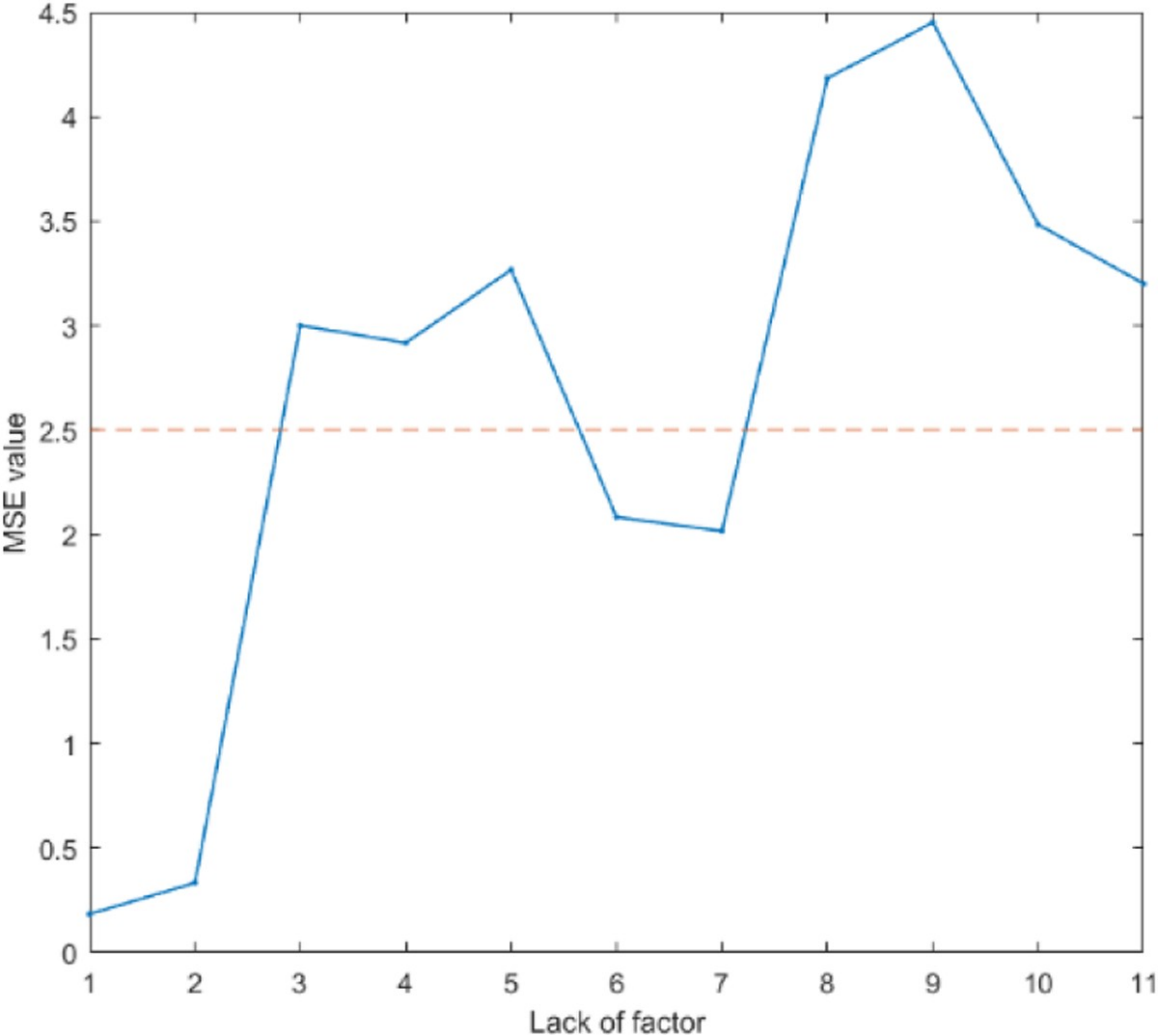
The mean square error value of the predicted results.

In this paper, we use the method of local sensitivity analysis to study the sensitivity of each factor. Fig 13 shows the mean square error value of the predicted results.

Among the 11 principal factors, 7 are more sensitive, including H1 skill level, H2 physical condition, H3 safety awareness, F2 maintenance and inspection, F3 facility instrument, G1 contraband goods, and G2 storage issues. The mean square error increased to 4.45 after reducing F2 factor, after reducing F3 factors mean square error increases to 4.1833, after reducing G1 factors mean square error increases to 3.4833, after reducing H3 factors mean square error increases to 3.2667, after reducing G2 factors mean square error increases to 3.2, after reducing H1 increase to 3 factors mean square error, after reducing the H2 factors mean square error increases to 2.9167.

### Risk factor analysis

#### Analysis of the principal factors leading to the accident

**Fig 14:**
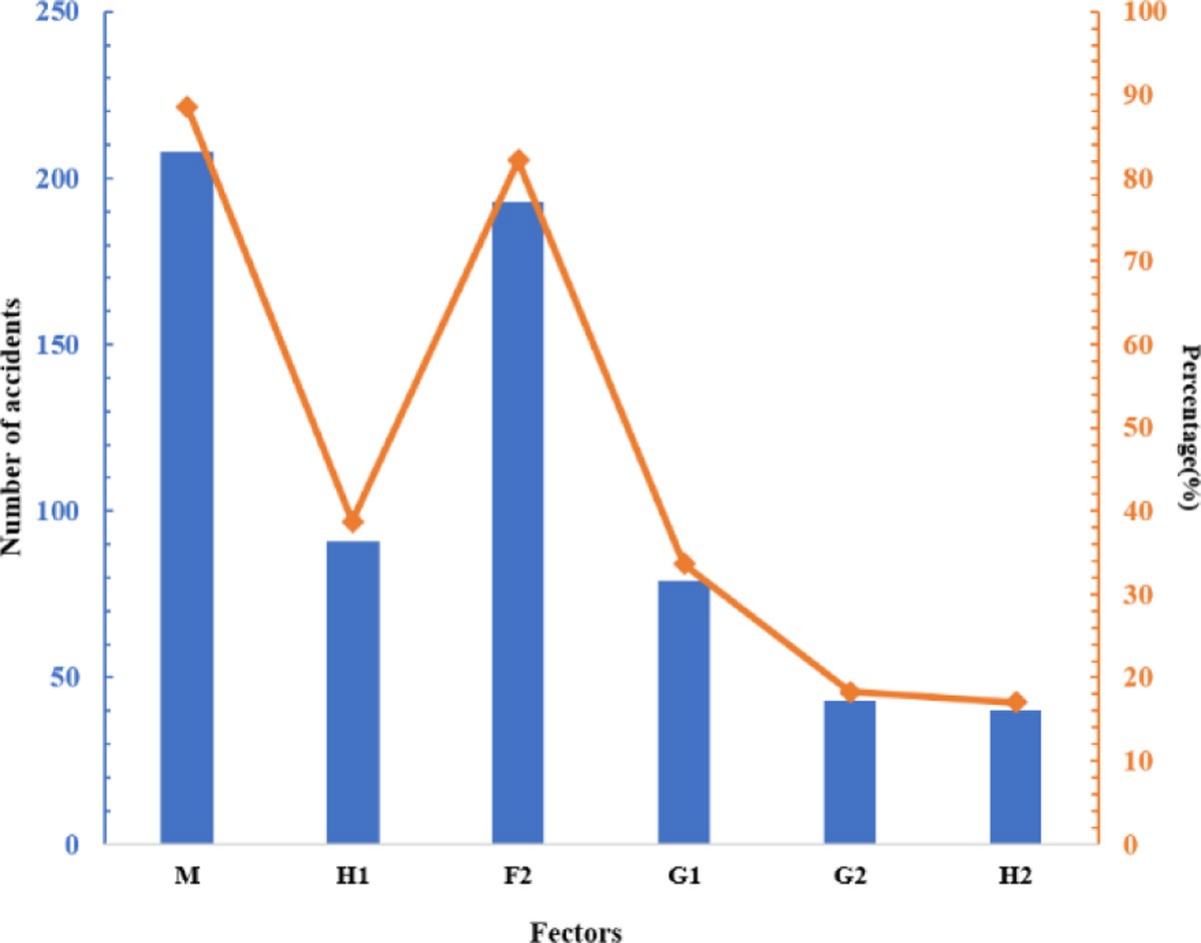
Risk factors occurred over 40 times.

**Fig 15:**
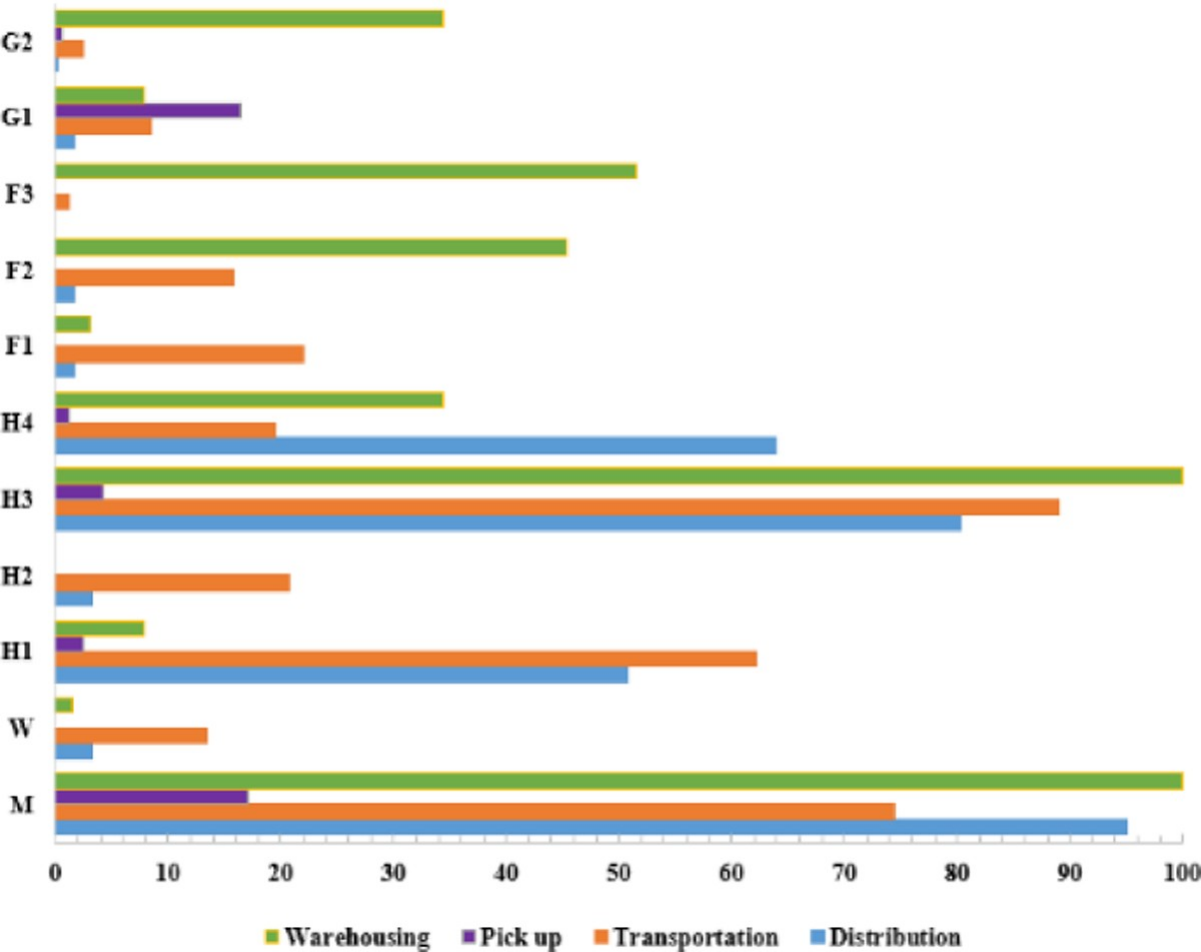
The occurrence frequency of relevant risk factors of accidents in various links of urban logistics.

In risk management, the effective measures to reduce the occurrence of accidents are to prevent the principal risk factors actively. We screened out the risk factors with over 40 occurrences, and Fig 14 shows the statistical results. Besides, Fig 15 shows the statistics of risk factors related to accidents in each link of urban logistics.

As shown in Fig 14, 88.51% of accidents are related to management factors (M), 82.13% are related to safety awareness factors (H3), and 38.72% are related to skill level factors (H1). These factors are calm to ignore in the actual security management. First of all, it is important to pay special attention to these indicators when hiring staff. Secondly, enterprises need to add safety skills training courses, and strengthen safety supervision. As shown in Fig 15, these three risk factors have a high correlation with accidents in each logistics link. Also, 63.93% of terminal distribution accidents, 34.38% of storage accidents, and 19.51% of transportation accidents are related to personnel quality (H4). It can be seen that logistics security management cannot ignore the attention to personnel quality factors, which need to be paid attention to when recruiting logistics staff.

#### Analysis of the combination of high-frequency factors leading to the accidents

**Fig 16:**
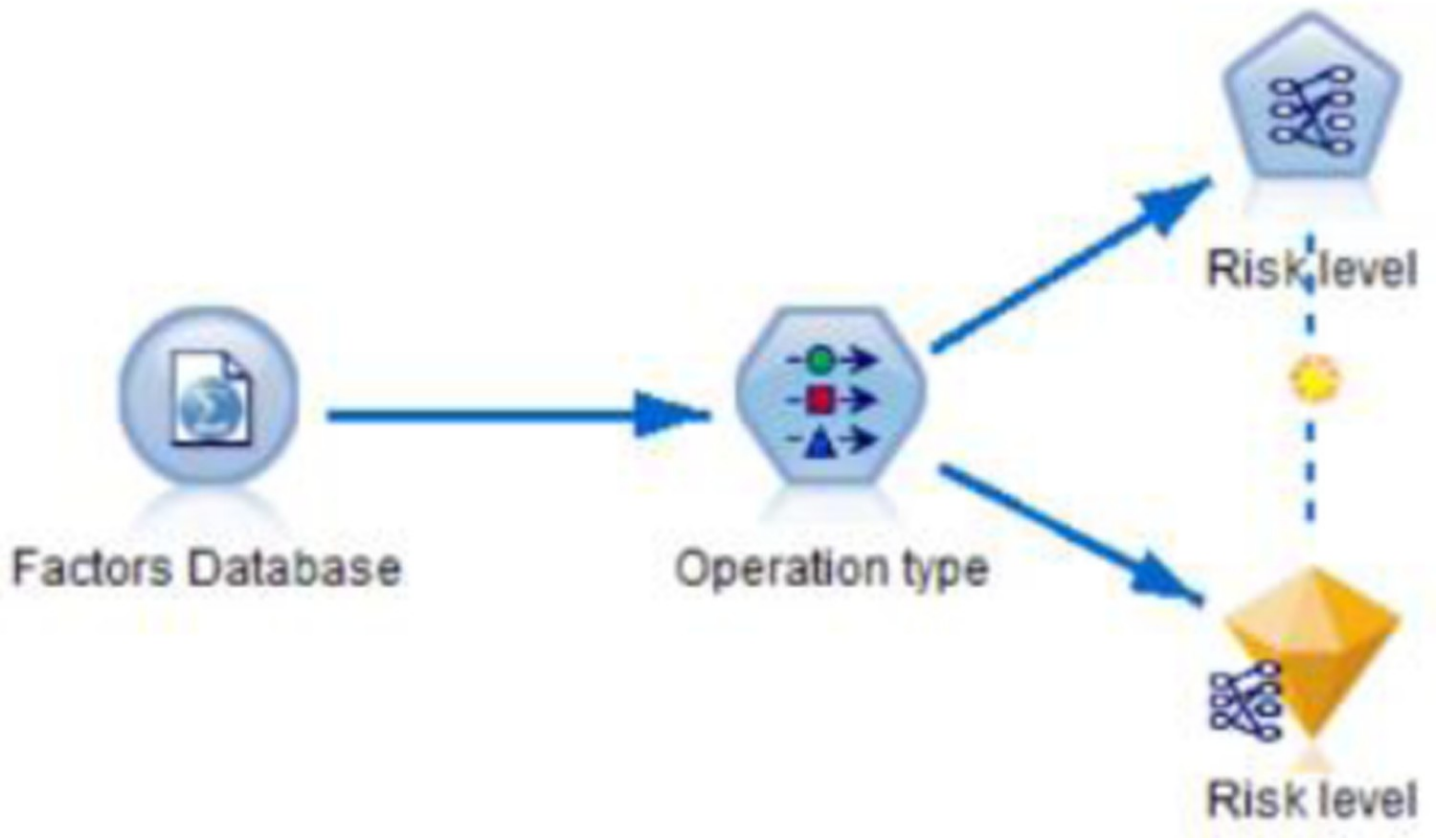
Associated data mining process of urban logistics to public accidents.

**Fig 17:**
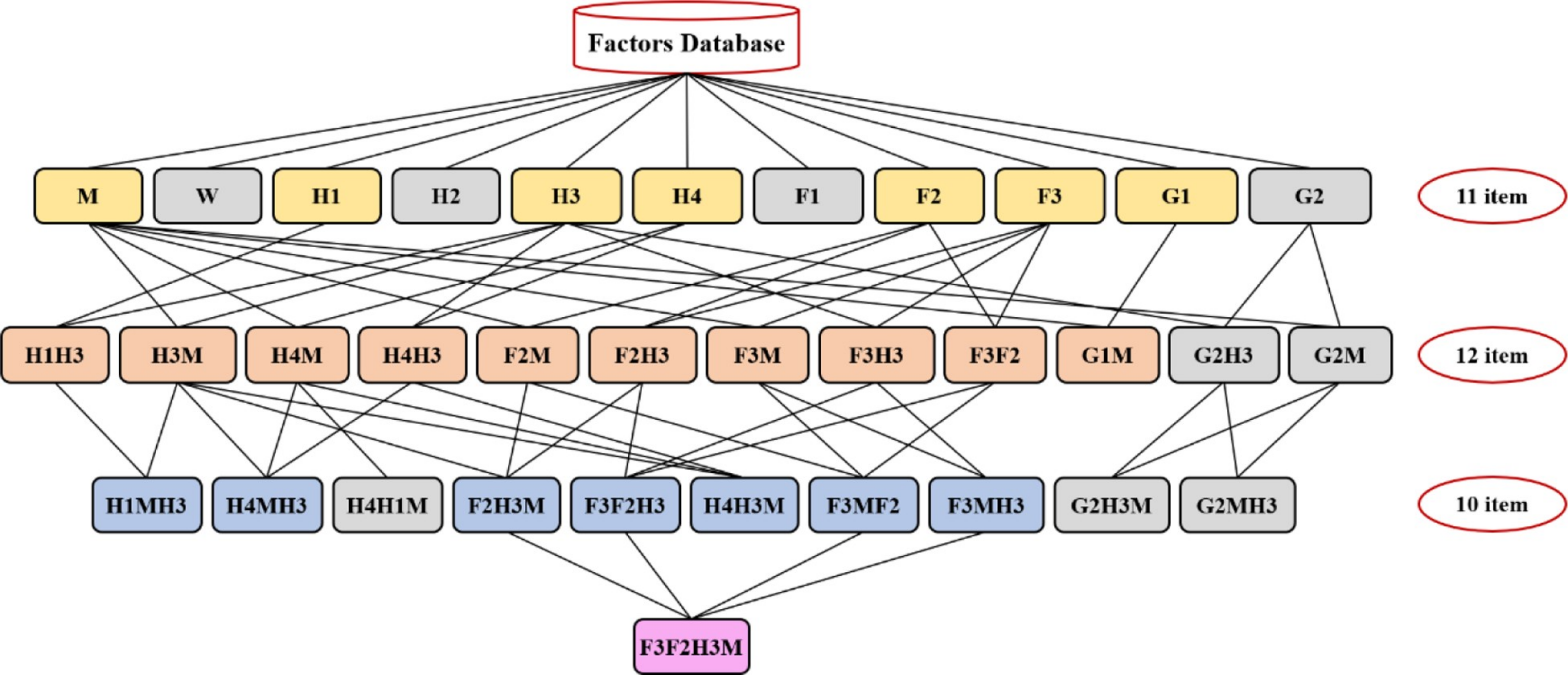
Topological relations of data mining for urban logistics to public accidents.

**Fig 18:**
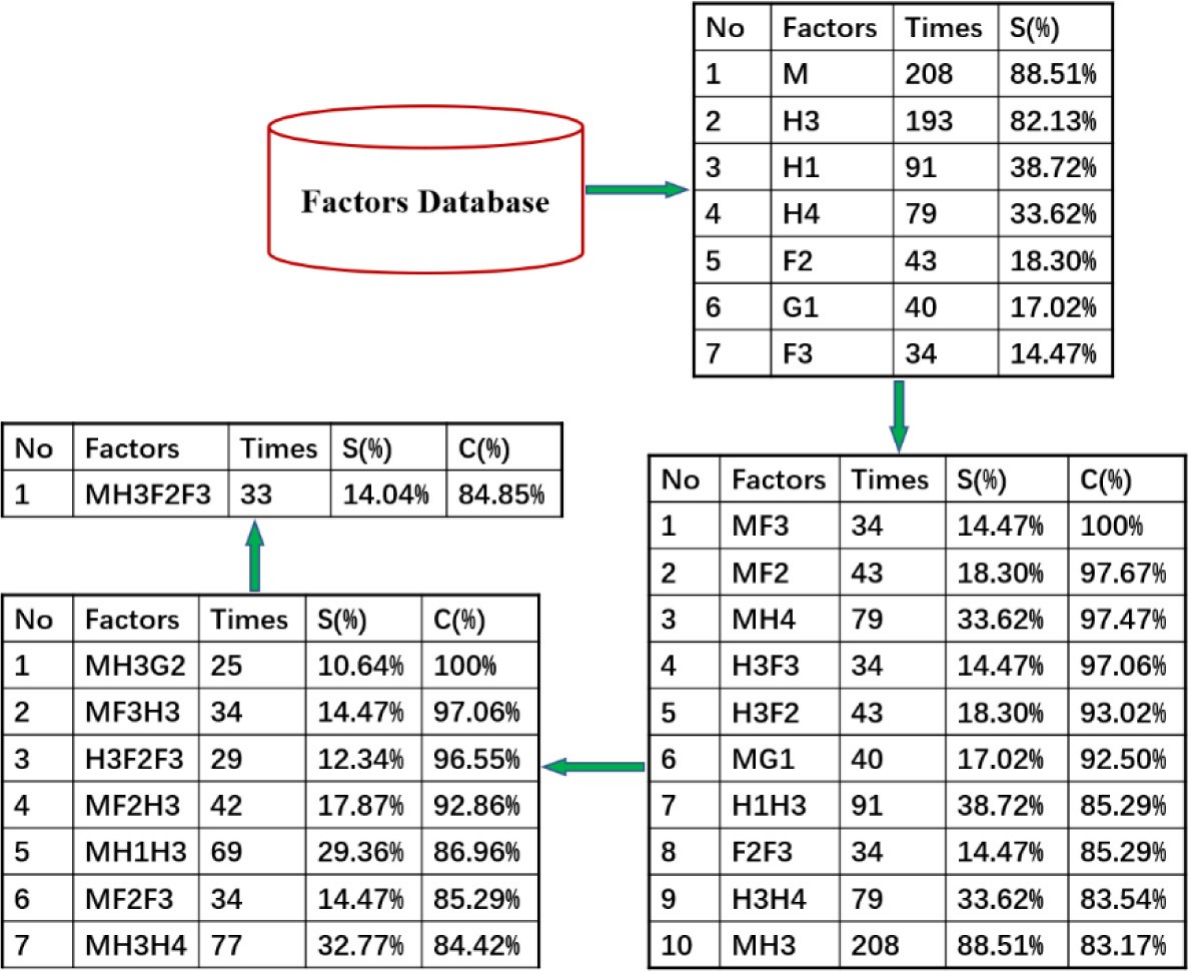
High-frequency factor combination of urban logistics to public accidents.

In safety management, the correlation between various risk factors is a common concern of managers. In this paper, the Apriori algorithm in IBM SPSS Modeler statistical modeling software is used to mine the high-frequency risk factors of public accidents caused by urban logistics. Fig 16 shows the mining process. Set the association rules as the minimum support is 14% and the minimum confidence is 80%. Fig 17 shows the data mining topology diagram, and it shows the mining results in Fig 18.

The data mining results show that, among the 235 public security accidents of urban logistics, there are 7 single factors closely related to the accidents, 10 two-factor combinations, 6 three-factor combinations, and 1 four-factor combination.

1. Single-factor. There are 208 public safety accidents of urban logistics related to improper management(M), accounting for 88.51% of the total incidents. There are 193 urban logistics public accidents related to safety awareness (H3), accounting for 82.13% of the total accidents. There are 91 public accidents of urban logistics related to the skill level of operators (H1), accounting for 38.72% of the total accidents. Other factors are analyzed in the same way.
2. Two-factor combination. There are 34 urban logistics public safety accidents related to the combination of management factors (M) and facility instrument (F3), accounting for 14.47% of the total accidents. Of all the accidents related to facility instrument (F3), 100% are related to management factors (M). There are 43 public accidents of urban logistics related to the combination of management factors (M), maintenance and inspection (F2), accounting for 18.30% of the total accidents. Of all the accidents related to maintenance and inspection (F2), 97.67% are related to management factors (M). So do other factors.
3. Three-factor combination. There are 34 urban logistics public safety accidents related to management factors (M), safety awareness (H3), and facility instrument (F3), accounting for 14.47% of the total accidents. There are 29 urban logistics public safety accidents related to safety awareness (H3), maintenance and inspection (F2), and facility instrument (F3), accounting for 12.34% of the total accidents. So do other factors.
4. Four-factor combination. There are 33 urban logistics public safety accidents related to management factors (M), safety awareness (H3), maintenance and inspection (F2), and facilities instrument (F3), accounting for 14.04% of the total accidents.

## Conclusion

This paper introduces a method of public security risk prediction and risk factor analysis of urban logistics. The main work and conclusions are as follows.

1. The prediction accuracy of the PSO-GRNN prediction model established in this paper for the public security risk level of urban logistics is 80%. Compared with BP neural network and GRNN, the PSO-GRNN model has the advantages of high prediction accuracy, high stability, and fast convergence.
2. Sensitivity analysis is conducted on urban logistics risk factors. The analysis results show that the sensitive factors are H1 skill level, H2 physical condition, H3 safety awareness, F2 maintenance and inspection, F3 facility instrument, G1 contraband goods, and G2 storage issues.
3. Apriori algorithm is used to mine the high-frequency risk factors of urban logistics public security. According to the association rules set in this paper, there are 10 two-factor high-frequency combinations, 6 three-factor high-frequency combinations, and 1 four-factor high-frequency combination.

In the practical application of urban logistics safety management, this study can predict the level of risk accidents and adopt the corresponding risk response mechanism and emergency plan. According to the combined analysis of high-frequency factors, managers can focus on high-frequency factors to reduce the risk of accidents. However, the model still needs to be improved and improved to make it more accurate in risk prediction and better applied to more disasters and accidents. We will consider the following aspects for in-depth study:

1. Add more detailed quantitative variables (such as cargo type) and qualitative variables (such as cargo weight).
2. The improved algorithm makes the model more suitable for the learning of missing value data, improves the accuracy of prediction, and is also more suitable for the prediction of various accidents.
3. Based on the study on the combination of high-frequency factors in this paper, the study on the coupling mechanism of factors will be further carried out to provide a more sufficient basis for managers to prevent and control urban logistics accidents.

## Supporting information

S1 Table Accident data set. (DOCX)
