# Supplementary material for: Risk prediction and risk factor analysis of urban logistics to public security based on PSO-GRNN algorithm
Source: PLoS One. 2020 Oct 5;15(10):e0238443. doi: 10.1371/journal.pone.0238443 (PMC7535052; doi:10.1371/journal.pone.0238443)
Supplement: S1 Table — (DOCX) [file pone.0238443.s001.docx]

S1 Table. Accident data set.

| M | W | H1 | H2 | H3 | H4 | F1 | F2 | F3 | G1 | G2 | L | M | W | H1 | H2 | H3 | H4 | F1 | F2 | F3 | G1 | G2 | L |
| --- | --- | --- | --- | --- | --- | --- | --- | --- | --- | --- | --- | --- | --- | --- | --- | --- | --- | --- | --- | --- | --- | --- | --- |
| 0.5 | 1 | 0.4 | 1 | 0 | 0.4 | 1 | 1 | 1 | 1 | 1 | 1 | 0.5 | 1 | 0.4 | 1 | 0.3 | 0.3 | 1 | 1 | 1 | 1 | 1 | 2 |
| 0.8 | 1 | 0 | 1 | 0 | 0.2 | 1 | 1 | 1 | 1 | 1 | 3 | 0.5 | 1 | 1 | 1 | 0.4 | 1 | 0.4 | 0.4 | 1 | 0.5 | 1 | 1 |
| 0.5 | 1 | 0.4 | 1 | 0 | 0.4 | 1 | 1 | 1 | 1 | 1 | 1 | 0.5 | 0.4 | 0.3 | 1 | 0.3 | 1 | 0.4 | 1 | 1 | 1 | 1 | 2 |
| 0.2 | 1 | 0 | 1 | 0 | 0.1 | 1 | 1 | 1 | 1 | 1 | 3 | 0.4 | 0.3 | 0.2 | 0.2 | 0.2 | 0.3 | 1 | 1 | 1 | 1 | 1 | 4 |
| 0.5 | 1 | 0.5 | 1 | 0 | 0.3 | 1 | 1 | 1 | 1 | 1 | 2 | 0.4 | 1 | 0.5 | 1 | 0 | 0.5 | 1 | 1 | 1 | 1 | 1 | 1 |
| 0.5 | 1 | 0.5 | 1 | 0 | 0.3 | 1 | 1 | 1 | 1 | 1 | 2 | 0.5 | 1 | 0.5 | 0.5 | 0.5 | 1 | 1 | 1 | 1 | 1 | 1 | 1 |
| 0.5 | 1 | 0.2 | 1 | 0.4 | 0.5 | 1 | 1 | 1 | 1 | 1 | 3 | 0.5 | 1 | 0.5 | 0.4 | 0.3 | 1 | 1 | 1 | 1 | 1 | 1 | 1 |
| 0.5 | 1 | 0.2 | 1 | 0.4 | 0.5 | 1 | 1 | 1 | 1 | 1 | 3 | 0.5 | 1 | 0.2 | 1 | 0.3 | 1 | 1 | 1 | 1 | 1 | 1 | 2 |
| 0.5 | 1 | 0.4 | 1 | 0.4 | 0.5 | 1 | 1 | 1 | 1 | 1 | 2 | 0.4 | 1 | 0.5 | 1 | 0.3 | 1 | 0.3 | 0.3 | 1 | 1 | 1 | 2 |
| 0.4 | 1 | 0.5 | 1 | 0.2 | 0.4 | 1 | 1 | 1 | 1 | 1 | 1 | 0.5 | 1 | 0.5 | 0.4 | 0.4 | 1 | 1 | 1 | 1 | 1 | 1 | 1 |
| 0.2 | 1 | 0.4 | 1 | 0.2 | 0.4 | 1 | 1 | 1 | 1 | 1 | 3 | 0.5 | 1 | 0.5 | 0.4 | 0.4 | 1 | 1 | 1 | 1 | 1 | 1 | 1 |
| 0.5 | 1 | 0.4 | 1 | 0.3 | 0.4 | 1 | 1 | 1 | 1 | 1 | 2 | 0.5 | 0.5 | 0.4 | 1 | 0.4 | 1 | 1 | 1 | 1 | 1 | 1 | 1 |
| 0.4 | 1 | 0.6 | 1 | 0.3 | 0.4 | 1 | 1 | 1 | 1 | 1 | 1 | 0.3 | 1 | 0.2 | 1 | 0.2 | 0.3 | 1 | 1 | 1 | 1 | 1 | 3 |
| 0.4 | 1 | 0.6 | 1 | 0.3 | 0.4 | 1 | 1 | 1 | 1 | 1 | 1 | 0.5 | 1 | 0.3 | 1 | 0.4 | 1 | 1 | 1 | 1 | 1 | 1 | 1 |
| 0.3 | 1 | 0.6 | 1 | 0.3 | 0 | 1 | 1 | 1 | 0.4 | 1 | 2 | 0.5 | 1 | 0.5 | 1 | 0.4 | 1 | 1 | 1 | 1 | 1 | 1 | 1 |
| 0.5 | 1 | 0 | 1 | 0 | 0.3 | 1 | 1 | 1 | 1 | 1 | 2 | 0.4 | 1 | 1 | 1 | 0.3 | 1 | 1 | 1 | 1 | 0.3 | 1 | 2 |
| 0.5 | 1 | 0.2 | 1 | 0.4 | 0 | 1 | 1 | 1 | 1 | 1 | 3 | 0.5 | 1 | 0.5 | 0.4 | 0.4 | 1 | 1 | 1 | 1 | 1 | 1 | 1 |
| 0.2 | 1 | 0.4 | 1 | 0.5 | 0.5 | 1 | 1 | 1 | 1 | 1 | 2 | 0.5 | 1 | 0.5 | 0.4 | 0.4 | 1 | 1 | 1 | 1 | 1 | 1 | 1 |
| 0.4 | 1 | 1 | 1 | 0 | 0.1 | 1 | 1 | 1 | 1 | 1 | 3 | 0.4 | 1 | 1 | 0 | 0.4 | 0.4 | 1 | 1 | 1 | 0.5 | 1 | 1 |
| 0.2 | 1 | 1 | 1 | 0 | 0.3 | 1 | 1 | 1 | 1 | 1 | 2 | 0.5 | 1 | 0.5 | 0.4 | 0.4 | 1 | 1 | 1 | 1 | 1 | 1 | 3 |
| 0.4 | 1 | 1 | 1 | 0 | 0.3 | 1 | 1 | 1 | 1 | 1 | 2 | 0.5 | 1 | 0.5 | 1 | 0.4 | 1 | 1 | 1 | 1 | 1 | 0.4 | 1 |
| 0.3 | 1 | 1 | 1 | 0 | 0.4 | 1 | 1 | 1 | 1 | 1 | 2 | 0.4 | 1 | 0.2 | 0.2 | 0.2 | 1 | 1 | 1 | 1 | 1 | 1 | 4 |
| 0.2 | 1 | 0.2 | 0.2 | 0.2 | 1 | 1 | 1 | 1 | 1 | 1 | 4 | 0.5 | 1 | 0.4 | 1 | 0.4 | 1 | 0.3 | 0.3 | 1 | 1 | 1 | 2 |
| 0.4 | 0.2 | 0.4 | 0.2 | 0.2 | 1 | 1 | 1 | 1 | 1 | 1 | 4 | 0.5 | 1 | 0.5 | 1 | 0.3 | 1 | 0.3 | 0.3 | 1 | 1 | 1 | 2 |
| 0.5 | 1 | 0.4 | 1 | 0.3 | 1 | 1 | 1 | 1 | 1 | 1 | 3 | 0.4 | 1 | 0.3 | 1 | 0.2 | 1 | 1 | 1 | 1 | 1 | 1 | 3 |
| 0.4 | 1 | 0.4 | 1 | 0.2 | 0.4 | 1 | 1 | 1 | 1 | 1 | 3 | 0.4 | 1 | 0.3 | 1 | 0.3 | 1 | 1 | 1 | 1 | 0.2 | 1 | 3 |
| 0.4 | 1 | 0.4 | 1 | 0.4 | 0.2 | 1 | 1 | 1 | 1 | 1 | 3 | 0.4 | 1 | 0.4 | 1 | 0.4 | 1 | 1 | 1 | 1 | 0.4 | 1 | 2 |
| 0.3 | 1 | 0.4 | 1 | 0.4 | 0.5 | 1 | 1 | 1 | 1 | 1 | 1 | 0.4 | 1 | 0.5 | 1 | 0.4 | 0.2 | 1 | 1 | 1 | 0.4 | 1 | 2 |
| 0.5 | 1 | 0.5 | 1 | 0.4 | 0.4 | 1 | 1 | 1 | 1 | 1 | 1 | 0.4 | 1 | 0.5 | 1 | 0.4 | 0.2 | 1 | 1 | 1 | 0.4 | 1 | 2 |
| 0.5 | 1 | 0.5 | 1 | 0.2 | 0.2 | 1 | 1 | 1 | 1 | 1 | 3 | 0.4 | 1 | 0.5 | 1 | 0.4 | 1 | 1 | 1 | 1 | 0.4 | 1 | 2 |
| 0.3 | 1 | 0.5 | 1 | 0.2 | 0.5 | 1 | 1 | 1 | 1 | 1 | 3 | 0.3 | 1 | 0.5 | 1 | 0.3 | 1 | 1 | 1 | 1 | 0.3 | 1 | 3 |
| 0.5 | 1 | 0.5 | 1 | 0.2 | 0.4 | 1 | 1 | 1 | 1 | 1 | 1 | 0.3 | 1 | 0.4 | 1 | 0.3 | 1 | 1 | 1 | 1 | 0.3 | 1 | 3 |
| 0.6 | 1 | 0.4 | 1 | 0.6 | 0.6 | 1 | 1 | 1 | 1 | 1 | 1 | 0.3 | 1 | 0.4 | 1 | 0.3 | 1 | 1 | 1 | 1 | 0.3 | 1 | 3 |
| 0.5 | 1 | 0.6 | 1 | 0.4 | 0.6 | 1 | 1 | 1 | 1 | 1 | 1 | 0.3 | 1 | 0.4 | 1 | 0.3 | 1 | 1 | 1 | 1 | 0.3 | 1 | 3 |
| 0.4 | 0.5 | 0.4 | 1 | 0.4 | 0.2 | 1 | 1 | 1 | 1 | 1 | 3 | 0.3 | 1 | 0.4 | 1 | 0.3 | 1 | 1 | 1 | 1 | 0.3 | 1 | 3 |
| 0.5 | 1 | 0.4 | 1 | 0.4 | 0.5 | 1 | 1 | 1 | 1 | 1 | 2 | 0.3 | 1 | 0.5 | 1 | 0.3 | 1 | 1 | 1 | 1 | 0.3 | 1 | 3 |
| 0.6 | 1 | 0.5 | 1 | 0.5 | 0 | 1 | 1 | 1 | 1 | 1 | 1 | 0.3 | 1 | 0.4 | 1 | 0.3 | 1 | 1 | 1 | 1 | 0.3 | 1 | 3 |
| 0.6 | 1 | 0.5 | 1 | 0.5 | 0 | 1 | 1 | 1 | 1 | 1 | 1 | 0.3 | 1 | 0.4 | 1 | 0.3 | 1 | 1 | 1 | 1 | 0.3 | 1 | 3 |
| 0.5 | 1 | 0.6 | 1 | 0.5 | 0.5 | 1 | 1 | 1 | 1 | 1 | 1 | 0.5 | 1 | 0.5 | 1 | 0.4 | 1 | 1 | 1 | 1 | 0.4 | 1 | 2 |
| 0.6 | 1 | 0.6 | 1 | 0.5 | 0.6 | 1 | 1 | 1 | 1 | 1 | 1 | 0.5 | 1 | 0.4 | 1 | 0.4 | 1 | 1 | 1 | 1 | 0.4 | 1 | 2 |
| 0.4 | 1 | 0.4 | 1 | 0.2 | 0.4 | 1 | 1 | 1 | 1 | 1 | 3 | 0.5 | 1 | 0.5 | 1 | 0.4 | 1 | 1 | 1 | 1 | 0.4 | 1 | 2 |
| 0.5 | 1 | 0.5 | 1 | 0.4 | 0 | 1 | 1 | 1 | 1 | 1 | 1 | 0.5 | 1 | 0.5 | 1 | 0.4 | 1 | 1 | 1 | 1 | 0.4 | 1 | 2 |
| 0.5 | 0.6 | 0.5 | 1 | 0.6 | 0.6 | 1 | 1 | 1 | 1 | 1 | 1 | 0.5 | 1 | 0.5 | 1 | 0.4 | 1 | 1 | 1 | 1 | 0.4 | 1 | 2 |
| 0.5 | 1 | 0.6 | 1 | 0.4 | 0 | 1 | 1 | 1 | 1 | 1 | 1 | 0.5 | 1 | 0.5 | 1 | 0.4 | 1 | 1 | 1 | 1 | 0.4 | 1 | 2 |
| 0.6 | 1 | 0.6 | 1 | 0.6 | 0 | 1 | 1 | 1 | 1 | 1 | 1 | 0.3 | 1 | 0.5 | 1 | 0.3 | 1 | 1 | 1 | 1 | 0.3 | 1 | 3 |
| 0.5 | 0.5 | 0.6 | 1 | 0.5 | 0.6 | 1 | 1 | 1 | 1 | 1 | 1 | 0.4 | 1 | 0.5 | 1 | 0.4 | 1 | 1 | 1 | 1 | 0.4 | 1 | 2 |
| 0.5 | 1 | 0.6 | 1 | 0.5 | 0.5 | 1 | 1 | 1 | 1 | 1 | 1 | 0.3 | 1 | 0.5 | 1 | 0.3 | 1 | 1 | 1 | 1 | 0.3 | 1 | 3 |
| 0.5 | 1 | 0.6 | 1 | 0.5 | 0.6 | 1 | 1 | 1 | 1 | 1 | 1 | 0.5 | 1 | 0.5 | 1 | 0.5 | 1 | 1 | 1 | 1 | 0.5 | 0.5 | 1 |
| 0.5 | 1 | 0.6 | 1 | 0.5 | 0.6 | 1 | 1 | 1 | 1 | 1 | 1 | 0.4 | 1 | 0.5 | 1 | 0.4 | 0.4 | 1 | 1 | 1 | 0.4 | 1 | 2 |
| 0.5 | 1 | 0.6 | 1 | 0.5 | 0.5 | 1 | 1 | 1 | 1 | 1 | 1 | 0.4 | 1 | 0.5 | 1 | 0.4 | 1 | 1 | 1 | 1 | 0.4 | 1 | 2 |
| 0.5 | 1 | 0.6 | 1 | 0.6 | 0.6 | 1 | 1 | 1 | 1 | 1 | 1 | 0.4 | 1 | 0.5 | 1 | 0.4 | 1 | 1 | 1 | 1 | 0.4 | 1 | 2 |
| 0.5 | 1 | 0.6 | 1 | 0.6 | 0.6 | 1 | 1 | 1 | 1 | 1 | 1 | 0.4 | 1 | 0.5 | 1 | 0.4 | 1 | 1 | 1 | 1 | 0.4 | 1 | 2 |
| 0.5 | 1 | 0.6 | 1 | 0.5 | 0.6 | 1 | 1 | 1 | 1 | 1 | 1 | 0.4 | 1 | 0.5 | 1 | 0.4 | 1 | 1 | 1 | 1 | 0.4 | 1 | 2 |
| 0.5 | 1 | 0.6 | 1 | 0.5 | 0.6 | 1 | 1 | 1 | 1 | 1 | 1 | 0.4 | 1 | 0.5 | 1 | 0.4 | 0.4 | 1 | 1 | 1 | 1 | 0.5 | 2 |
| 0.5 | 1 | 0.6 | 1 | 0.5 | 0.6 | 1 | 1 | 1 | 1 | 1 | 1 | 0.2 | 1 | 1 | 1 | 0.3 | 1 | 1 | 1 | 1 | 1 | 0.3 | 4 |
| 0.5 | 1 | 0.6 | 1 | 0.5 | 0.5 | 1 | 1 | 1 | 1 | 1 | 1 | 0.5 | 1 | 1 | 1 | 0.5 | 1 | 1 | 1 | 1 | 1 | 0.5 | 1 |
| 0.5 | 1 | 0.6 | 1 | 0.6 | 0.5 | 1 | 1 | 1 | 1 | 1 | 1 | 0.1 | 1 | 0.2 | 1 | 0.2 | 1 | 1 | 1 | 1 | 0.2 | 0.2 | 5 |
| 0.5 | 1 | 0.6 | 1 | 0.6 | 0.5 | 1 | 1 | 1 | 1 | 1 | 1 | 0.1 | 0.3 | 1 | 1 | 0.2 | 1 | 1 | 1 | 1 | 0.2 | 0.2 | 5 |
| 0.5 | 1 | 0.6 | 1 | 0.6 | 0.5 | 1 | 1 | 1 | 1 | 1 | 1 | 0.3 | 1 | 1 | 1 | 0.3 | 1 | 1 | 1 | 1 | 0.4 | 0.4 | 3 |
| 0.5 | 1 | 0.6 | 1 | 0.6 | 0.5 | 1 | 1 | 1 | 1 | 1 | 1 | 0.4 | 1 | 1 | 1 | 0.4 | 1 | 1 | 1 | 1 | 0.5 | 0.4 | 2 |
| 0.7 | 1 | 0.6 | 1 | 0.6 | 0 | 1 | 1 | 1 | 1 | 1 | 1 | 0.4 | 1 | 1 | 1 | 0.4 | 1 | 1 | 1 | 1 | 1 | 0.4 | 2 |
| 0.4 | 1 | 0.5 | 0.4 | 0.2 | 1 | 1 | 1 | 1 | 1 | 1 | 3 | 0.2 | 1 | 1 | 1 | 0.3 | 0.3 | 1 | 1 | 0.2 | 1 | 0.3 | 4 |
| 0.6 | 1 | 0 | 0.4 | 0.4 | 1 | 1 | 1 | 1 | 1 | 1 | 1 | 0.3 | 1 | 1 | 1 | 0.3 | 1 | 1 | 1 | 0.3 | 1 | 0.5 | 3 |
| 0.6 | 1 | 0.5 | 0.4 | 0.2 | 1 | 1 | 1 | 1 | 1 | 1 | 3 | 0.4 | 1 | 1 | 1 | 0.4 | 1 | 1 | 1 | 1 | 1 | 0.4 | 2 |
| 0.4 | 1 | 0.4 | 1 | 0.2 | 1 | 1 | 1 | 1 | 1 | 1 | 3 | 0.4 | 1 | 1 | 1 | 0.4 | 1 | 1 | 1 | 1 | 1 | 0.4 | 2 |
| 0.3 | 1 | 0.3 | 1 | 0.1 | 1 | 0.4 | 1 | 1 | 1 | 1 | 4 | 0.3 | 1 | 1 | 1 | 0.3 | 0.3 | 1 | 1 | 1 | 1 | 0.4 | 3 |
| 0.5 | 0.3 | 0.4 | 1 | 0.5 | 1 | 1 | 1 | 1 | 1 | 1 | 3 | 0.4 | 1 | 1 | 1 | 0.4 | 1 | 1 | 1 | 1 | 1 | 0.4 | 2 |
| 0.4 | 1 | 0.3 | 0.4 | 0.3 | 1 | 1 | 1 | 1 | 1 | 1 | 4 | 0.3 | 1 | 1 | 1 | 0.3 | 1 | 1 | 1 | 1 | 1 | 0.4 | 3 |
| 0.5 | 1 | 0.4 | 1 | 0.2 | 1 | 0.4 | 1 | 1 | 1 | 1 | 3 | 0.4 | 1 | 1 | 1 | 0.4 | 1 | 1 | 0.4 | 0.4 | 1 | 1 | 2 |
| 0.4 | 1 | 0.6 | 1 | 0.4 | 1 | 0.4 | 0.4 | 1 | 1 | 1 | 2 | 0.4 | 1 | 1 | 1 | 0.4 | 0.4 | 1 | 1 | 0.4 | 1 | 1 | 2 |
| 0.4 | 1 | 0.5 | 1 | 0.3 | 1 | 0.3 | 0.3 | 1 | 0.3 | 0.5 | 4 | 0.4 | 1 | 1 | 1 | 0.4 | 0.4 | 1 | 1 | 1 | 1 | 1 | 2 |
| 0.5 | 1 | 0.4 | 0.3 | 0.3 | 1 | 1 | 1 | 1 | 1 | 1 | 3 | 0.4 | 1 | 0.4 | 1 | 0.4 | 0.4 | 1 | 1 | 1 | 1 | 1 | 2 |
| 0.4 | 1 | 0.2 | 0 | 0.3 | 1 | 1 | 1 | 1 | 1 | 1 | 3 | 0.2 | 1 | 1 | 1 | 0.3 | 1 | 1 | 0.2 | 0.2 | 1 | 0.3 | 4 |
| 0.5 | 1 | 0.5 | 0.4 | 0.5 | 1 | 1 | 1 | 1 | 1 | 1 | 1 | 0.2 | 1 | 1 | 1 | 0.3 | 1 | 1 | 0.2 | 0.2 | 1 | 0.3 | 4 |
| 0.5 | 0.2 | 0.3 | 1 | 0.3 | 1 | 1 | 1 | 1 | 1 | 1 | 4 | 0.2 | 1 | 1 | 1 | 0.3 | 1 | 1 | 0.2 | 0.2 | 1 | 0.3 | 4 |
| 0.5 | 1 | 1 | 1 | 0.5 | 1 | 1 | 1 | 1 | 0.4 | 0.5 | 1 | 0.2 | 1 | 1 | 1 | 0.3 | 1 | 1 | 0.2 | 0.2 | 1 | 0.3 | 4 |
| 0.5 | 0.4 | 1 | 1 | 0.5 | 1 | 1 | 1 | 1 | 0.4 | 1 | 1 | 0.2 | 1 | 1 | 1 | 0.3 | 1 | 1 | 0.2 | 0.2 | 1 | 0.3 | 4 |
| 0.5 | 1 | 0.2 | 1 | 0.3 | 1 | 1 | 1 | 1 | 1 | 1 | 3 | 0.3 | 1 | 0.4 | 1 | 0.3 | 0.4 | 1 | 1 | 0.4 | 1 | 1 | 3 |
| 0.4 | 0.4 | 0.4 | 1 | 0.4 | 1 | 0.2 | 0.3 | 1 | 1 | 1 | 3 | 0.2 | 1 | 1 | 1 | 0.3 | 1 | 1 | 0.2 | 0.2 | 1 | 0.3 | 4 |
| 0.4 | 1 | 0.5 | 1 | 0.5 | 1 | 0.4 | 0.4 | 1 | 1 | 1 | 2 | 0.1 | 1 | 1 | 1 | 0.1 | 0.1 | 1 | 1 | 1 | 1 | 0.2 | 5 |
| 0.6 | 1 | 0.3 | 1 | 0.4 | 1 | 1 | 1 | 1 | 1 | 1 | 2 | 0.3 | 1 | 1 | 1 | 0.3 | 1 | 1 | 0.3 | 0.3 | 1 | 1 | 3 |
| 0.3 | 1 | 0.4 | 1 | 0.2 | 0.3 | 1 | 1 | 1 | 1 | 1 | 4 | 0.3 | 1 | 1 | 1 | 0.3 | 0.2 | 1 | 1 | 1 | 1 | 1 | 3 |
| 0.3 | 1 | 0.3 | 1 | 0.2 | 0.2 | 1 | 1 | 1 | 1 | 1 | 3 | 0.4 | 1 | 1 | 1 | 0.4 | 1 | 1 | 0.4 | 0.4 | 1 | 1 | 2 |
| 0.3 | 1 | 0.3 | 1 | 0.2 | 0.2 | 0.3 | 1 | 1 | 1 | 1 | 3 | 0.4 | 1 | 1 | 1 | 0.4 | 0.3 | 1 | 1 | 1 | 1 | 1 | 2 |
| 0.5 | 1 | 0.2 | 1 | 0.2 | 0.2 | 1 | 1 | 1 | 1 | 1 | 3 | 0.4 | 1 | 1 | 1 | 0.4 | 0.3 | 1 | 1 | 0.3 | 1 | 0.3 | 3 |
| 0.5 | 1 | 0.5 | 1 | 0.4 | 1 | 1 | 1 | 1 | 1 | 1 | 1 | 0.4 | 1 | 1 | 1 | 0.4 | 0.3 | 1 | 1 | 0.3 | 1 | 0.3 | 3 |
| 0.4 | 1 | 0.5 | 1 | 0.5 | 0.5 | 1 | 1 | 1 | 1 | 1 | 1 | 0.2 | 1 | 0.3 | 1 | 0.3 | 1 | 1 | 0.2 | 0.2 | 1 | 1 | 4 |
| 0.4 | 1 | 0.5 | 1 | 0.5 | 0.6 | 1 | 1 | 0.5 | 1 | 1 | 1 | 0.2 | 1 | 1 | 1 | 0.3 | 1 | 1 | 0.2 | 0.2 | 1 | 1 | 4 |
| 0.6 | 1 | 0.4 | 1 | 0.5 | 1 | 1 | 1 | 1 | 1 | 1 | 1 | 0.1 | 1 | 1 | 1 | 0.2 | 1 | 1 | 0.1 | 0.1 | 1 | 1 | 5 |
| 0.4 | 1 | 0.5 | 1 | 0.4 | 0.4 | 1 | 1 | 1 | 1 | 1 | 2 | 0.1 | 1 | 1 | 1 | 0.1 | 1 | 1 | 0.2 | 0.1 | 1 | 0.2 | 5 |
| 0.5 | 1 | 0.4 | 1 | 0.4 | 1 | 1 | 1 | 1 | 1 | 1 | 2 | 0.1 | 1 | 1 | 1 | 0.1 | 0.1 | 1 | 1 | 1 | 1 | 1 | 5 |
| 0.5 | 0.4 | 0.4 | 1 | 0.5 | 1 | 1 | 1 | 1 | 1 | 1 | 1 | 0.1 | 1 | 1 | 1 | 0.1 | 1 | 1 | 0.2 | 0.1 | 1 | 0.2 | 5 |
| 0.5 | 1 | 0.4 | 0.4 | 0.4 | 1 | 1 | 1 | 1 | 1 | 1 | 2 | 0.3 | 1 | 1 | 1 | 0.3 | 1 | 1 | 0.3 | 0.3 | 1 | 1 | 3 |
| 0.5 | 1 | 0.2 | 1 | 0.4 | 1 | 1 | 1 | 1 | 1 | 1 | 3 | 0.3 | 1 | 1 | 1 | 0.3 | 1 | 1 | 0.3 | 0.3 | 1 | 1 | 3 |
| 0.3 | 0.2 | 0.2 | 1 | 0.2 | 1 | 0.2 | 1 | 1 | 1 | 1 | 5 | 0.3 | 1 | 1 | 1 | 0.3 | 1 | 1 | 0.3 | 0.3 | 1 | 1 | 3 |
| 0.6 | 1 | 0.4 | 1 | 0.5 | 1 | 1 | 1 | 1 | 1 | 1 | 1 | 0.3 | 1 | 1 | 1 | 0.3 | 0.3 | 1 | 1 | 1 | 1 | 1 | 3 |
| 0.5 | 1 | 0.6 | 0.4 | 0.4 | 1 | 1 | 1 | 1 | 1 | 1 | 1 | 0.4 | 1 | 1 | 1 | 0.4 | 0.3 | 1 | 1 | 1 | 1 | 0.4 | 2 |
| 0.5 | 1 | 0.6 | 1 | 0.5 | 1 | 1 | 1 | 1 | 1 | 1 | 1 | 0.4 | 1 | 1 | 1 | 0.4 | 1 | 1 | 0.4 | 0.4 | 1 | 1 | 2 |
| 0.4 | 1 | 0.5 | 1 | 0.5 | 1 | 0.4 | 0.4 | 0.4 | 1 | 1 | 2 | 0.4 | 1 | 1 | 1 | 0.4 | 1 | 1 | 0.4 | 0.4 | 1 | 1 | 2 |
| 0.4 | 1 | 1 | 1 | 0.5 | 1 | 1 | 1 | 1 | 0.3 | 0.3 | 2 | 0.4 | 1 | 1 | 1 | 0.4 | 0.3 | 1 | 0.4 | 0.4 | 1 | 1 | 2 |
| 0.4 | 1 | 1 | 1 | 0.4 | 1 | 0.3 | 0.3 | 1 | 1 | 1 | 2 | 0.4 | 1 | 1 | 1 | 0.4 | 0.3 | 1 | 0.4 | 1 | 1 | 1 | 2 |
| 0.3 | 1 | 0.2 | 1 | 0.2 | 0.2 | 1 | 1 | 1 | 1 | 1 | 4 | 0.4 | 1 | 1 | 1 | 0.4 | 1 | 1 | 0.4 | 0.4 | 1 | 1 | 2 |
| 0.4 | 0.5 | 0.3 | 1 | 0.2 | 1 | 0.4 | 1 | 1 | 1 | 1 | 3 | 0.3 | 1 | 1 | 1 | 0.3 | 1 | 1 | 0.2 | 0.2 | 1 | 1 | 3 |
| 0.4 | 0.4 | 0.3 | 1 | 0.2 | 1 | 1 | 1 | 1 | 0.5 | 1 | 3 | 0.3 | 1 | 0.3 | 1 | 0.3 | 0.2 | 1 | 1 | 1 | 1 | 1 | 3 |
| 0.5 | 1 | 0.5 | 0.4 | 0.3 | 1 | 0.3 | 1 | 1 | 1 | 1 | 3 | 0.4 | 1 | 0.4 | 1 | 0.4 | 1 | 1 | 0.3 | 0.3 | 1 | 1 | 2 |
| 0.5 | 1 | 0.5 | 0.3 | 0.2 | 1 | 1 | 1 | 1 | 1 | 1 | 3 | 0.1 | 1 | 1 | 1 | 0.1 | 1 | 1 | 0.2 | 0.1 | 1 | 0.1 | 5 |
| 0.5 | 1 | 0.3 | 1 | 0.2 | 1 | 1 | 1 | 1 | 1 | 1 | 3 | 0.1 | 1 | 0.2 | 1 | 0.1 | 1 | 1 | 0.2 | 0.1 | 1 | 0.1 | 5 |
| 0.4 | 0.2 | 0.2 | 1 | 0.2 | 1 | 1 | 1 | 1 | 1 | 1 | 4 | 0.1 | 1 | 1 | 1 | 0.1 | 1 | 1 | 0.2 | 0.1 | 1 | 0 | 5 |
| 0.4 | 1 | 0.2 | 1 | 0.2 | 1 | 1 | 1 | 1 | 1 | 1 | 3 | 0.1 | 1 | 0.2 | 1 | 0.1 | 1 | 1 | 0.2 | 0.1 | 1 | 0.1 | 5 |
| 0.5 | 0.5 | 0.3 | 1 | 0.2 | 1 | 1 | 1 | 1 | 1 | 1 | 3 | 0.1 | 1 | 1 | 1 | 0.1 | 0.1 | 1 | 1 | 1 | 1 | 0.1 | 5 |
| 0.5 | 1 | 1 | 0.5 | 0.5 | 0.5 | 1 | 1 | 1 | 1 | 1 | 1 | 0.2 | 1 | 1 | 1 | 0.3 | 1 | 0.2 | 1 | 1 | 1 | 0.2 | 4 |
| 0.4 | 1 | 0.3 | 1 | 0.2 | 0.2 | 1 | 1 | 1 | 1 | 1 | 3 | 0.4 | 1 | 1 | 1 | 0.4 | 1 | 0.3 | 0.4 | 1 | 1 | 1 | 2 |
| 0.4 | 1 | 0.3 | 1 | 0.2 | 0.3 | 1 | 1 | 1 | 1 | 1 | 3 | 0.4 | 1 | 1 | 1 | 0.4 | 1 | 1 | 1 | 1 | 0.3 | 0.3 | 2 |
| 0.4 | 0.3 | 0.3 | 1 | 0.2 | 1 | 0.3 | 0.2 | 1 | 1 | 1 | 4 | 0.2 | 1 | 1 | 1 | 0.3 | 1 | 1 | 0.2 | 0.2 | 1 | 0.2 | 4 |
| 0.3 | 1 | 0.2 | 1 | 0.2 | 0.2 | 0.3 | 0.3 | 1 | 1 | 1 | 4 | 0.3 | 1 | 1 | 1 | 0.3 | 1 | 1 | 1 | 1 | 0.3 | 0.3 | 3 |
| 0.3 | 1 | 0.2 | 1 | 0.2 | 0.2 | 1 | 1 | 1 | 1 | 1 | 4 | 0.3 | 1 | 1 | 1 | 0.3 | 1 | 1 | 0.3 | 0.3 | 1 | 1 | 3 |
| 0.5 | 1 | 0.5 | 0.2 | 0.2 | 0.4 | 1 | 1 | 1 | 1 | 1 | 3 | 0.2 | 1 | 1 | 1 | 0.3 | 0.2 | 1 | 1 | 1 | 1 | 0.2 | 4 |
| 0.5 | 1 | 0.5 | 1 | 0.4 | 1 | 1 | 1 | 1 | 0.5 | 1 | 1 |  |  |  |  |  |  |  |  |  |  |  |  |
